# PTPN9 dephosphorylates IGF1R^Y1165/1166^ and alleviates IGF1R-mediated resistance to tyrosine kinase inhibitor in cholangiocarcinoma

**DOI:** 10.1186/s13046-025-03594-2

**Published:** 2025-11-22

**Authors:** Jia-ming Hu, Hui-qiang Liu, Ming-hui Zhang, Tian-li Chen, An-da Shi, Qiang Gao, Yun-jia Liu, Xin Wang, Kai-yang Sun, Jian Deng, Yun-fei Xu, Chang Pan, Kang-shuai Li, Zong-li Zhang

**Affiliations:** 1 Department of General Surgery, Qilu Hospital, Cheeloo College of Medicine, Shandong University, 107 Wenhua Xi Road, Jinan, Shandong 250012 P.R. China; 2 Department of Emergency Medicine, Qilu Hospital of Shandong University, 107 Wenhua Xi Road, Jinan, 250012 P.R. China; 3 Shandong Provincial Clinical Research Center for Emergency and Critical Care Medicine, Institute of Emergency and Critical Care Medicine of Shandong University, Chest Pain Center, Qilu Hospital of Shandong University, 107 Wenhua Xi Road, Jinan, 250012 China; 4 Key Laboratory of Emergency and Critical Care Medicine of Shandong Province, Key Laboratory of Cardiopulmonary-Cerebral Resuscitation Research of Shandong Province, Qilu Hospital of Shandong University, 107 Wenhua Xi Road, Jinan, 250012 China

**Keywords:** Cholangiocarcinoma, Surufatinib, IGF1R, PTPN9, CAF

## Abstract

Cholangiocarcinoma (CCA) is an aggressive malignancy with limited therapeutic options. Increasing evidence suggests that a subset of CCA patients can benefit from multiple tyrosine kinase inhibitors (mTKIs) such as surufatinib. Biomarker studies that can identify potential responders to mTKI therapy are crucial for improving treatment outcomes. In this study, we examined the expression of protein tyrosine phosphatase non-receptor type 9 (PTPN9) in CCA patients treated with surufatinib and analyzed its association with treatment response. Immunoprecipitation coupled with mass spectrometry (IP-MS) was performed to identify novel PTPN9 substrates. Orthotopic mouse models, biochemical assays, structural analyses, and functional experiments were conducted to validate these novel findings. Single-cell RNA sequencing, ELISA, and immunohistochemistry were utilized to investigate microenvironmental tumor-promoting factors.

CCA patients with surufatinib non-response exhibited significantly decreased PTPN9 expression compared to that response to surufatinib. PTPN9 knockdown in orthotopic models led to reduced sensitivity to surufatinib. IGF1R was identified as a new substrate of PTPN9, which preferably to dephosphorylate IGF1R at Y1166. In clinical tissues, PTPN9 expression was inversely correlated with IGF1R Y1165/1166 phosphorylation. Establishment of a surufatinib-resistant CCA cell line further confirmed decreased PTPN9 expression and elevated IGF1R signaling. In vivo blockade of IGF1R signaling significantly enhanced surufatinib sensitivity. Mechanistically, crystal structure analysis revealed Tyr333 and Asp335 as key PTPN9 residues interacting with IGF1R; mutation of these residues restored IGF1R signaling and abolished the tumor-suppressive effect of PTPN9. Furthermore, cancer-associated fibroblasts (CAFs) were identified as the major source of IGF1 in CCA microenvironment, essential for IGF1R-driven tumor progression.

In summary, the PTPN9–IGF1R axis plays a pivotal role in modulating mTKI sensitivity and tumor progression in CCA. This axis serves as a promising biomarker for identifying potential mTKI beneficiaries and represents a potential therapeutic target to enhance mTKI efficacy and overcome resistance.

**Supplementary Information:**

The online version contains supplementary material available at 10.1186/s13046-025-03594-2.

## Introduction

Cholangiocarcinoma (CCA) is a highly aggressive malignancy of the biliary tract characterized by limited therapeutic options and poor clinical outcomes [1]. Despite rising global incidence and mortality, effective targeted treatments remain scarce [2, 3]. Current standard chemotherapy regimens, primarily gemcitabine combined with cisplatin, yield disappointing long-term survival rates, emphasizing a critical unmet need for novel therapeutic strategies [4]. Genomic profiling has revealed potential therapeutic targets such as FGFR2 fusions and IDH1/2 mutation; however, the mutation rates were relatively low [5]. Although no multi-target tyrosine kinase inhibitors (mTKIs) have yet received FDA approval, small-scale clinical observations indicate that agents such as surufatinib, lenvatinib, and anlotinib can confer clinical benefit to CCA patients when administered as monotherapy or in combination regimens [6–10]. However, a subset of cholangiocarcinoma patients exhibits intrinsic resistance to mTKIs [9, 10]. In currently observed mTKI-immunotherapy combination strategies, alterations in the tumor immune infiltrate landscape correlate with therapeutic response [11]. Notably, research focusing on CCA tumor cell-intrinsic alterations as mediators of mTKI resistance or predictive biomarkers for mTKI treatment response remains largely unexplored.

Primary and bypass resistance mechanisms significantly limit the therapeutic efficacy and durability of tyrosine kinase inhibitors (TKIs). Primary resistance frequently arises from pre-existing or early emerging RTK pathway alterations that diminish tumor dependency on the targeted receptor. In EGFR-mutant non-small cell lung cancer, third-generation EGFR-TKIs inevitably select for on-target mutations (e.g., C797S) and bypass signaling (e.g., MET/ERBB), undermining long-term control [12, 13]. In renal cell carcinoma, resistance to VEGFR-directed TKIs such as sunitinib is linked to mesenchymal/AXL programs and integrin–focal-adhesion signaling that promote drug tolerance and progression [14, 15]. In FGFR2-altered cholangiocarcinoma, clinical sequencing at progression frequently reveals polyclonal secondary FGFR2 mutations and pathway rewiring, limiting the durability of FGFR inhibitors [16]. In hepatocellular carcinoma (HCC), bypass activation of EGFR, VEGFR, and FGFR is implicated in mediating resistance to lenvatinib [17, 18]. Furthermore, bypass signaling activation via IGF1R is recognized as a mechanism of resistance to mTKIs across multiple cancer types [19–23]. The IGF1R pathway is increasingly implicated in CCA pathobiology [24, 25]. IGF1R is a transmembrane tyrosine kinase activated by IGF-1 or IGF-2, triggering downstream signaling cascades such as IRS-PI3K-AKT and RAS-RAF-ERK that promote cell proliferation, survival, and migration [26]. While IGF1R signaling normally regulates tissue growth, its dysregulation frequently contributes to oncogenesis, positioning IGF1R as a potential prognostic marker and therapeutic target in CCA [25].

Protein tyrosine phosphorylation levels are antagonistically regulated by protein tyrosine kinases (PTKs) and protein tyrosine phosphatases (PTPs). Consequently, resistance mechanisms to mTKIs may be associated with PTP dysfunction. Notably, previous studies revealed that at least one PTP mutation occurs in 51.6% of intrahepatic cholangiocarcinoma (iCCA) patients [27]. Multiple investigations have demonstrated that functional alterations of PTPs significantly correlate with CCA prognosis, tumor cell behavior, and chemotherapy resistance [28, 29]. Our recent study further uncovered that PTPN9 exhibits differential expression in CCA, where its high expression serves as a favorable prognostic biomarker [30]. Mechanistically, PTPN9 negatively regulates FGFR2 phosphorylation via the sec14p-APCP1-FGFR2 interaction, thereby influencing the therapeutic response to Pemigatinib. However, whether PTPN9 contributes to mTKI resistance in CCA remains elusive.

In this study, we identified that high PTPN9 expression was correlated with response to mTKI inhibitor in iCCA and found that IGF1R is a mediator of PTPN9 related mTKI resistance. PTPN9 acts as a PTP that dephosphorylates p-IGF1R^Y1166^. Crystal structures and enzymatic analysis was then performed to assess the molecular mechanism while clinical relevance of PTPN9-IGF1R interaction was also investigated. Single cell RNA analysis and subsequent co-culture system was established to analyze the CAF-tumor cell interaction.

## Methods

### Crystallization and data collection

For crystallization, His-PTPN9-C515S/D470A protein (concentration at 15 mg/ml) was mixed with p-IGF1R^Y1165/1166^ peptide (IYETDpYpYRKGGK) with molar ratio as 1:3 in buffer A (pH 7.2, 20 mM HEPES, 350 mM NaCl, and 1 mM DTT). 1 µl mixed protein was blended with 1 µl buffer B (pH 6.4, 20% PEG 4000, 0.2 M KSCN, 10% ethylene glycol, 0.1 M bis-tris propane) at 4 °C for 3 days before crystals appear. The cubic crystals were preserved in liquid nitrogen after dipped in storage buffer (buffer B supplemented with 10% glycerol). The data were collected at Shanghai Synchrotron Radiation Facility beamline BL17U1 using 0.98 A X-ray wavelength and analyzed by HKL2000.

### Structural determination and refinement

The crystals of PTPN9-p-IGF1R^Y1165/1166^ peptides belong to the P1 space group. Molecular replacement of the PTPN9-peptide complex was performed with Phaser in the CCP4 software package, with PTPN9 catalytic domain (PDB code: 2PA5, water deleted) as the initial search model. Further refinements were carried out using the PHENIX program with iterative manual building in COOT.

### Protein expression and purification

The wide type and mutant proteins of GST-PTPN9 were expressed in BL21-DE3 E. coli in the presence of 0.4 mM IPTG (Sangon Biotech, B541007) for 16 h at 25 °C. After lysis and centrifugation using Sorvall LYNX 4000 High speed centrifuge (Thermo Fisher Scientific, 46910, US), the proteins were purified by binding with GST-Sepharose for 2 h and eluted by GSH. All protein were Feezed with liquid nitrogen and stored in a −80 °C refrigerator.

## Results

### PTPN9-IGF1R mediates TKI resistance in CCA

Our previous study has revealed that high PTPN9 expression correlates with favorable prognosis of CCA, however, the number of iCCA patients was relatively low and multivariate analysis of prognostic factors was not performed. To further validate the prognostic role of PTPN9 in CCA, we have developed a new cohort containing 161 iCCA patients, 128 pCCA patients and 138 dCCA patients. Due to partial chip detachment, the expression level of PTPN9 could be evaluated in 153 iCCA, 117 pCCA and 121 dCCA (Fig. S1A). Consistent with previous findings, we observed that high PTPN9 expression was associated with a favorable prognosis in iCCA, pCCA, and dCCA (Fig. S1B). High PTPN9 expression correlates with smaller tumor size in iCCA, higher tumor differentiation and lower T stage in pCCA and higher tumor differentiation in dCCA (Table S1). Multivariate analysis confirmed that intratumoral PTPN9 expression was an independent prognostic factor of iCCA, pCCA, and dCCA highlighting the significant role of PTPN9 in the progression of CCA (Table S2).

**Fig. 1:**
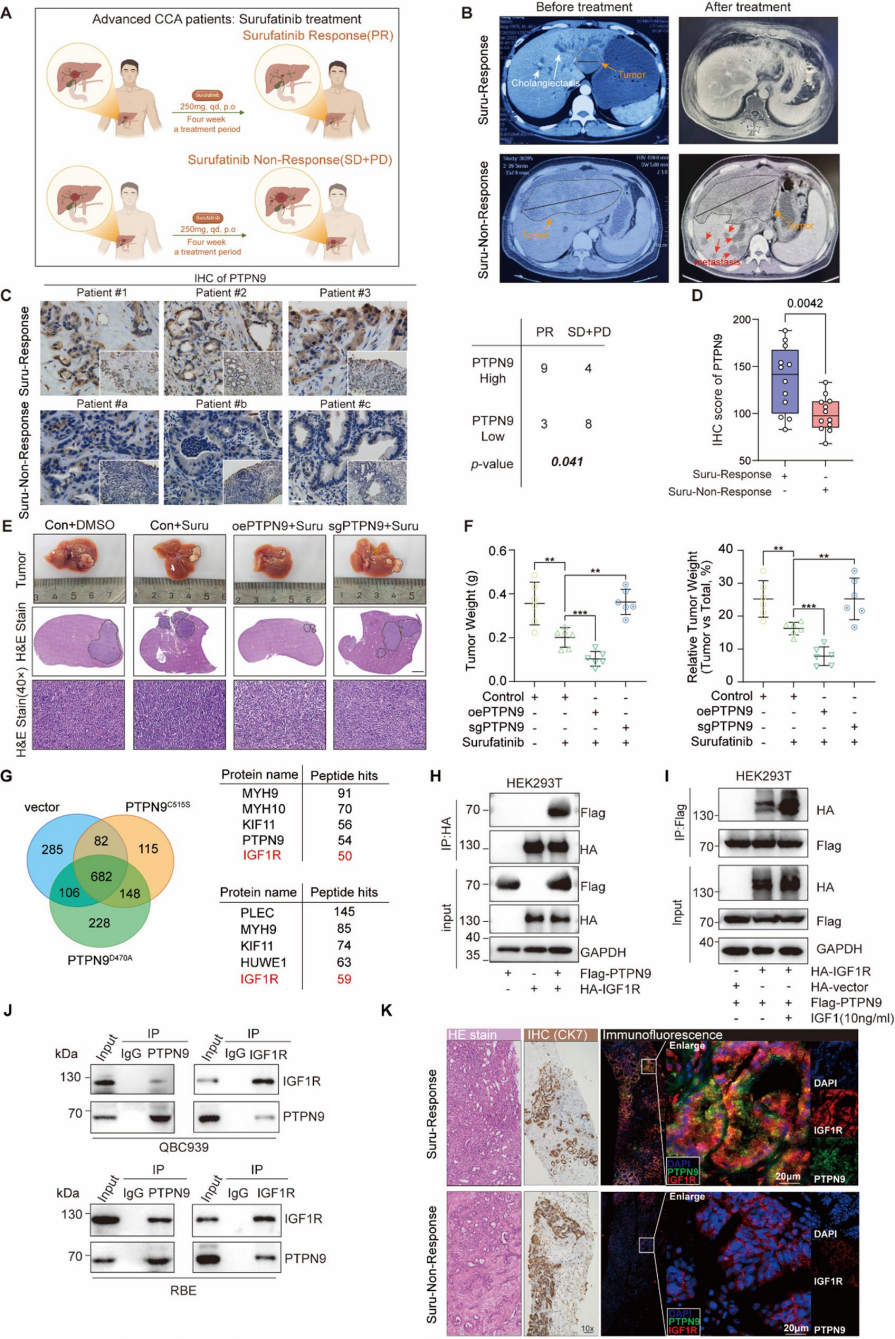
PTPN9 enhances surufatinib sensitivity by interacting with IGF1R in cholangiocarcinoma.**A** Clinical schematic of advanced CCA patients treated with surufatinib (250 mg/day, orally, for 4 weeks). **B** Representative CT and MRI images before and after surufatinib treatment in response and non-response patients. **C** Left: Representative immunohistochemical staining of PTPN9 in tumor tissues from surufatinib-response and -non-response patients. Right: Contingency table comparing treatment response between patients with low and high PTPN9 expression. Scale bar: 50 μm. **D** Quantification of PTPN9 IHC scores in surufatinib response vs. non-response tumor tissues. **E** Representative tumor images and H&E‑stained liver sections from orthotopic models (*n* = 6 per group) receiving DMSO, surufatinib, oePTPN9 with surufatinib or sgPTPN9-1 with surufatinib. Tumor regions are outlined with dotted lines. Scale bars: 2 mm (middle panels), 25 μm (lower panels). **F** Tumor weight (left) and relative tumor weight (right) in the orthotopic model. **G** Left: Venn diagram of proteins identified by LC–MS/MS after immunoprecipitation of empty vector, Flag-tagged PTPN9^C515S^ and PTPN9^D470A^ inactive mutants from HEK293T cells. Right: The special and top 5 interactors of PTPN9^C515S^ and the interactors of PTPN9^D470A^. **H** Co-immunoprecipitation (Co-IP) assay showing the interaction between Flag-tagged PTPN9 and HA-tagged IGF1R in HEK293T cells. **I** Anti-Flag immunoprecipitation and subsequent western blot was performed in Flag-tagged PTPN9 and HA-IGF1R/vector overexpressed HEK293T cells. **J** Reciprocal endogenous co-immunoprecipitation of PTPN9 and IGF1R was performed in QBC-939 (upper) and RBE (lower) CCA cell lines. **K** Representative images of H&E staining, CK7 immunohistochemistry, and immunofluorescence for PTPN9 (green) and IGF1R (red) in surufatinib-response (top) and -non-response (bottom) CCA samples. Nuclei were counterstained with DAPI (blue). Scale bars, 20 μm Data are from at least three independent experiments and are presented as mean ± SD or representative images. Statistical analyses were performed using Chi-square test (**C**) and unpaired t-test (**D**, **F**). Significance indicators: **P < 0.01, ***P < 0.001

Recently, we have observed primary resistance in a cohort of patients receiving surufatinib treatment, a muti-tyrosine kinase inhibitor. To further clarify the potential resistance mechanism, we analyzed the PTPN9 expression in tissue sections from CCA patients who exhibited non-response or response to surufatinib treatment (Fig. 1A, B), and found that PTPN9 expression was significantly lower in surufatinib-non-response CCA tumor tissues, indicating a potential role of PTPN9 in surufatinib resistance in CCA (Fig. 1C, D). To further confirm the role of PTPN9 in mediating surufatinib resistance, we established an orthotopic CCA model in C57BL/6 mice using PTPN9 overexpressed or knocked down LD1 murine CCA cell line and treated with surufatinib by intragastric administration (Fig. S1C). Interestingly, we found that surufatinib treatment inhibited tumor growth, overexpression of PTPN9 exhibited higher inhibit effect, whereas knockout of PTPN9 markedly exacerbated tumor growth (Fig. 1E, F). Surufatinib is a multiple tyrosine kinase inhibitor of VEGFR, FGFR and CSF1R and the PTPN9 induced surufatinib resistance could not due to the known PTPN9-FGFR2 interaction. We therefore hypothesized existence of unknown PTPN9 substrates which could be tyrosine kinases which may not be affected by surufatinib. To validate this hypothesis, we first performed immunoprecipitation coupled with mass spectrometry (IP-MS) using two trapping mutants of PTPN9, namely Flag-tagged PTPN9^C515S^ or Flag-tagged PTPN9^D470A^, to identify potential substrate of PTPN9 in HEK293T cells. Among the 148 proteins identified both in Flag-tagged PTPN9^C515S^ and Flag-tagged PTPN9^D470A^ but not in Flag-vector group, IGF1R is an important receptor and listed in fifth by peptide hits (Fig. 1G). Then, we analyzed IGF1R expression levels in cholangiocarcinoma using data from the TCGA-CHOL database, focusing on the RTK family. A volcano plot revealed that IGF1R was markedly upregulated in tumor tissues and ranked among the top RTKs (Fig. S1D), expression values for IGF1R were subsequently extracted and presented as a bar plot (Fig. S1E). Subsequently, to determine the appropriate cell lines for experimental validation, we examined IGF1R and PTPN9 expression in several commonly used cholangiocarcinoma cell lines and a normal biliary epithelial cell line. (Fig. S1F). Among them, QBC939 and RBE cells exhibited relatively high IGF1R and low PTPN9 expression. In addition, QBC939 and RBE were established separately from intrahepatic and extrahepatic cholangiocarcinoma. Therefore, QBC-939 and RBE cell lines were selected for subsequent experiments. We further confirmed the interaction between PTPN9 and IGF1R by both exogenous (Fig. 1H, I) and endogenous (Fig. 1J) Co-IP assays. Meanwhile, we performed immunofluorescence staining for PTPN9 and IGF1R both in surufatinib-response and -non-response CCA patient tissues. In rensponse tissues, PTPN9 and IGF1R exhibited clear colocalization, consistent with the regulatory role of PTPN9 in dephosphorylating IGF1R. Interestingly, in non-response tissues, the colocalization was markedly reduced, suggesting that low expression of PTPN9 in non-response tumors decreases its spatial interaction with IGF1R, potentially contributing to sustained IGF1R activation (Fig. 1K).

### PTPN9 interacts and dephosphorylates IGF1R^Y1165/1166^ in CCA

**Fig. 2:**
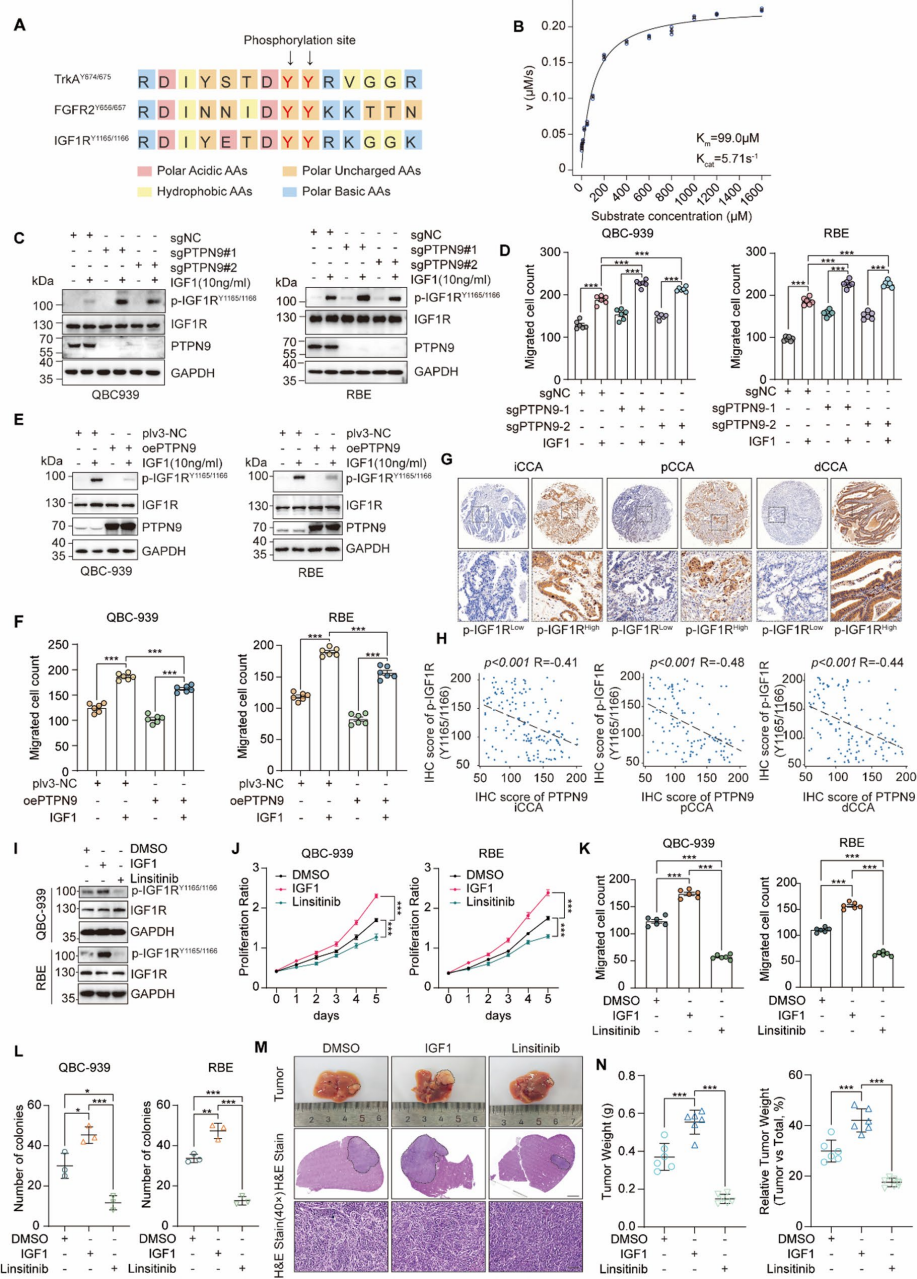
PTPN9 modulates phosphorylation level of IGF1R^Y1165/1166^ which is significant in tumor growth and CCA progression. **A** Alignment of activation loop phosphotyrosine motifs in TrkA, FGFR2, and IGF1R. **B** Michaelis–Menten kinetics of PTPN9 toward a phospho-IGF1R^Y1165/1166^ peptide. **C** Left (QBC-939) and right (RBE): CRISPR/Cas9 knockout of PTPN9 augments 10 ng/mL IGF1-induced IGF1R^Y1165/1166^ phosphorylation compared with sgNC. **D** Transwell migration assays indicate enhanced motility in sgPTPN9 cells upon IGF1 stimulation in QBC-939 and RBE lines. **E** oePTPN9 suppresses IGF1-stimulated IGF1R phosphorylation. **F** Over-expression of PTPN9 attenuates IGF1-induced migration in QBC-939 and RBE cells. **G** Representative IHC images showing high- and low- p-IGF1R expression in iCCA, pCCA and dCCA tumor tissues. Scale bar: 25 μm. **H** Scatter plots show the inverse correlation between IHC scores of PTPN9 and p-IGF1R^Y1165/1166^ in iCCA, pCCA, and dCCA tissue samples. **I** Immunoblot of QBC-939 and RBE cells treated with DMSO, IGF1 (10 ng/mL) or Linsitinib (10 µM) shows modulation of IGF1R^Y1165/1166^ phosphorylation; GAPDH serves as loading control. **J** CCK-8 assays show that IGF1 (10 ng/mL) stimulation enhances proliferation of QBC-939 and RBE cells, whereas Linsitinib (10 µM) treatment suppresses this effect. **K**, **L** IGF1 (10 ng/mL) promotes migration (**K**), and colony formation (**L**) in QBC-939 and RBE cells, while Linsitinib (10 µM) reverses these effects. **M** Orthotopic liver CCA model in C57BL/6 mice (*n* = 6 per group) treated with DMSO, IGF1(15 mg/kg, i.p.) or Linsitinib (30 mg/kg, i.p.). Scale bars: 2 mm (middle panels), 25 μm (lower panels). (**N**) Tumor weight (left) and relative tumor weight (right) in the orthotopic model Data are from at least three independent experiments and are presented as mean ± SD or representative images. Statistical analyses were performed using two-way ANOVA (**J**), Pearson correlation analysis (**H**) and unpaired t-test (**D**, **F**, **K**, **L**, **N**). Significance indicators: *P < 0.05, **P < 0.01, ***P < 0.001

Bioinformatics analysis indicated a high sequence homology around IGF1R phosphorylation sites Y1165/1166 compared with known dephosphorylation substrates of PTPN9, including TrkA^Y674/675^ and FGFR2^Y656/657^ (Fig. 2A). Enzymatic interaction between PTPN9 and p-IGF1R^Y1165/1166^ was confirmed by a Michaelis-Menten kinetics assay. Kinetic analysis demonstrated a clear enzyme-substrate relationship between PTPN9 and p-IGF1R^Y1165/1166^ (Fig. 2B).

To further demonstrate that p-IGF1R^Y1165/1166^ is negatively regulated by PTPN9, we established stable PTPN9 knockout (sgPTPN9) and overexpression (oePTPN9) cell lines derived from QBC-939 and RBE cells, validated by Western blotting (Fig. S2A, B). While alterations in PTPN9 expression did not influence total IGF1R protein levels, knockout of PTPN9 notably increased, whereas overexpression decreased IGF1-stimulated IGF1R^Y1165/1166^ phosphorylation (Fig. 2C). Functional assays demonstrated that PTPN9 knockout remarkably enhanced cell proliferation, colony formation, migration, and invasion capabilities upon IGF1 stimulation. (Fig. 2D, Fig. S2C-E). Ectopic expression of PTPN9 both in QBC939 and RBE cells suppressed this functional role of IGF1 treatment (Fig. 2F, Fig. S2F-H). Next, tumor tissue microarrays from patients were analyzed for p-IGF1R^Y1165/1166^, followed by correlation analysis with PTPN9 expression. A significant inverse correlation was observed between p-IGF1R^Y1165/1166^ levels and PTPN9 expression (Fig. 2G, H). Moreover, survival analysis based on p-IGF1R expression revealed that patients with high p-IGF1R levels had poorer prognosis compared to those with low expression (Fig.S2I, J).

To investigate the functional role of IGF1R activation in vitro, we selected IGF1 as an agonist and linsitinib as an IGF1R inhibitor. Western blot analysis showed that IGF1 and linsitinib did not alter total IGF1R expression but modulated phosphorylation levels of IGF1R at Y1165/1166, with IGF1 increasing and linsitinib decreasing phosphorylation (Fig. 2I). Functional assays demonstrated that IGF1 treatment significantly promoted cell proliferation, colony formation, migration, and invasion capabilities (Fig. 2J-L, Fig.S2K, L), whereas linsitinib had inhibitory effects in both cell lines.

Stable oeIGF1R and sgIGF1R QBC-939 and RBE cell lines were constructed and validated by western blot analysis (Fig. S3A). Overexpression of IGF1R enhanced proliferation, colony formation, migration, and invasion capabilities, and these effects were elevated by IGF1 treatment (Fig. S3B-E). Conversely, knockout of IGF1R impaired these cellular functions, which could be partially rescued by IGF1 treatment (Fig. S3F-I).

To assess in vivo relevance, we established an orthotopic liver cholangiocarcinoma model in C57BL/6 mice by liver injection of LD1 cells. Mice were administered with IGF1, or linsitinib intraperitoneally. 12 days post-injection, IGF1 significantly promoted liver tumor growth, while linsitinib markedly suppressed tumor development compared to the control group (Fig. 2M). Tumor weight and tumor-to-total liver weight ratio were both increased by IGF1 treatment and significantly reduced by linsitinib treatment compared to controls (Fig. 2N).

In summary, our results demonstrate that PTPN9 negatively regulates IGF1R phosphorylation at Y1165/1166 through direct enzymatic interaction. Modulating PTPN9 expression influences IGF1-induced phosphorylation, affecting cholangiocarcinoma cell proliferation, migration, and invasion. Furthermore, IGF1-driven IGF1R activation promotes tumor progression both in vitro and in vivo, while IGF1R inhibition via linsitinib effectively reduces these oncogenic effects, highlighting the significance of the PTPN9–IGF1R signaling axis in cholangiocarcinoma.

### IGF1R bypass activation promotes TKI resistance in CCA

**Fig. 3:**
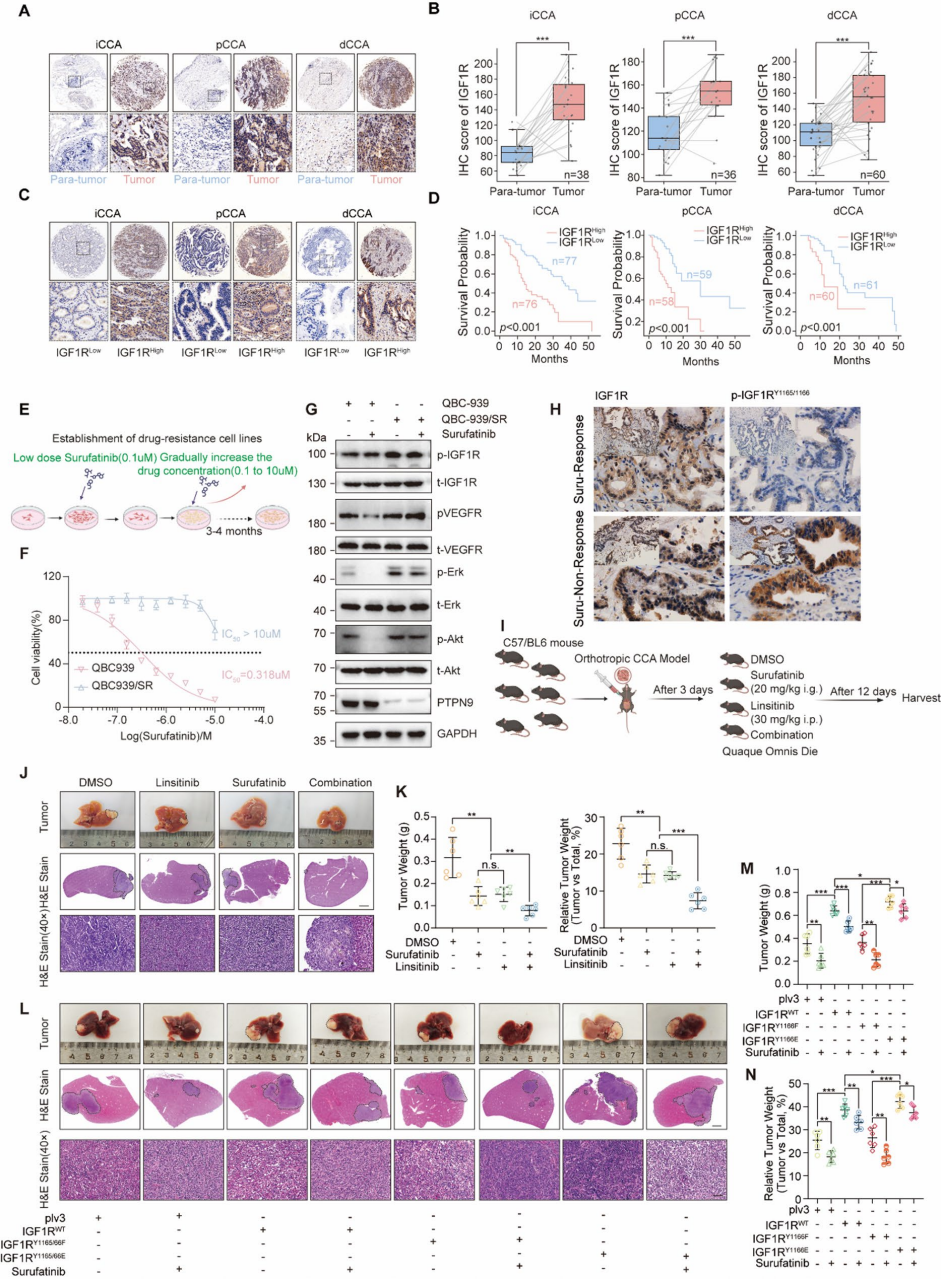
Elevated IGF1R expression promotes surufatinib resistance and indicates poor prognosis in cholangiocarcinoma.**A** Representative IHC images showing IGF1R expression in para-tumor tissue and high- and low-IGF1R expression iCCA, pCCA and dCCA tumor tissues. Scale bar: 25 μm. **B** Box-and-whisker plots comparing IGF1R IHC scores in paired tumor and para-tumor tissues from iCCA, pCCA and dCCA cohorts. **C** Representative IHC images showing high- and low- IGF1R expression in iCCA, pCCA and dCCA tumor tissues. Scale bar: 25 μm. **D** Kaplan–Meier overall-survival analysis was performed to analyze the prognosis role of IGF1R expression in iCCA, pCCA, and dCCA. **E** Schematic for establishment of QBC‑939/SR via stepwise drug exposure. **F** Cell viability assays revealed significantly reduced sensitivity to surufatinib in QBC‑939/SR compared to parental controls. **G** Western blot analysis of QBC‑939 and QBC‑939/SR cells with or without surufatinib treatment. **H** Representative immunohistochemical staining showing IGF1R and p-IGF1R expressions in surufatinib-response and -non-response cholangiocarcinoma samples. Scale bars: 50 μm. **I** Experimental setup of orthotopic CCA model in C57BL/6 mice treated with DMSO, surufatinib (20 mg/kg, i.g.), linsitinib (30 mg/kg, i.p.), or combination. **J** Representative tumor images and H&E staining from orthotopic CCA models (*n* = 6 per group) treated with DMSO, surufatinib, linsitinib or combination therapy. Tumor areas are outlined with dotted lines. Scale bars: 2 mm (middle panels), 25 μm (lower panels). **K** Tumor weight (left) and relative tumor weight ratio (right) in the orthotopic model. **L** Representative images of liver tumors and corresponding H&E-stained sections from orthotopic CCA models (*n* = 6 per group) expressing IGF1R^WT^ or IGF1R^Y1165/66F^, IGF1R^Y1165/66E^ with or without surufatinib treatment. Scale bars: 2 mm (middle panels), 25 μm (lower panels). **M**, **N** Quantification of tumor weight (**M**) and relative tumor weight (**N**) Data are from at least three independent experiments and are presented as mean ± SD or representative images. Statistical analyses were performed using paired t-test (**B**), log-rank test (**D**) and unpaired t‑test (**K**, **M**, **N**). Significance indicators: n.s., not significant, *P < 0.05, **P < 0.01, ***P < 0.001

Immunohistochemical staining was performed on a tissue microarray (TMA) comprising paired tumor and adjacent non-tumorous tissues from patients with iCCA, pCCA, and dCCA, which revealed significantly elevated IGF1R expression in tumor tissues across all subtypes (Fig. 3A, B). In a separate TMA cohort containing tumor tissues from CCA patients, IGF1R expression levels were evaluated and patients were stratified into IGF1R^High^ and IGF1R^Low^ groups based on the median IHC score. Kaplan-Meier survival analysis revealed that patients with high IGF1R expression had significantly worse overall survival compared to those with low IGF1R expression across all three CCA subtypes (Fig. 3C, D). In addition, multivariate Cox regression analysis revealed that IGF1R was an unfavorable prognostic factor (Fig. S4A-C, Table S3, 4).

Since PTPN9 was lower expression in surufatinib-non-response CCA tissues and PTPN9 negatively regulates IGF1R phosphorylation at Y1165/1166, we further evaluated that whether IGF1R bypass activation might be one of the potential mechanisms of resistance to surufatinib in CCA patients. Therefore, we established surufatinib-resistant cholangiocarcinoma cells (QBC-939/SR) using a gradual dose-escalation method (Fig. 3E). Cell viability assays demonstrated that QBC-939/SR cells exhibited significantly higher resistance to surufatinib compared to parental cells (Fig. 3F). To elaborate the molecular mechanism of IGF1R mediated surufatinib resistance, we detected the activation of IGF1R and PI3K-Akt and MEK-Erk pathways in QBC-939/SR cell or parental cells with or without surufatinib treatment. Western blot analyses revealed increased phosphorylation of IGF1R at residues Y1165/1166 in QBC-939/SR cells, accompanied by elevated activation of downstream signaling pathways, including phosphorylated Akt and Erk, notably, PTPN9 expression in QBC-939/SR cells was unchanged with or without surufatinib treatment (Fig. 3G). Furthermore, total IGF1R expression did not differ between surufatinib-response and -non-response CCA tissues, whereas p-IGF1R^Y1165/1166^ was increased in the non-response group (Fig. 3H, Fig. S4D). Consistently, cellular proliferation, colony formation, and migration/invasion assays indicated enhanced tolerance to surufatinib in QBC-939/SR cells relative to parental cells (Fig. S4E, F).

In addition, cell viability assays also revealed reduced survival in QBC-939/SR cells treated with linsitinib compared to DMSO controls (Fig. S4G). Furthermore, combined treatment with surufatinib and linsitinib markedly suppressed colony formation in QBC-939 cells compared to either drug alone (Fig. S4H). To further evaluate this therapeutic approach in vivo, we established an orthotopic cholangiocarcinoma model in C57BL/6 mice and administered surufatinib, linsitinib, or a combination of both drugs (Fig. 3I). After 12 days, tumors from combination-treated mice were markedly smaller than those treated with single agents (Fig. 3J), tumor weights and tumor-to-total liver weight ratios were significantly reduced in single-drug treatment groups compared to the control group, with the combination therapy group demonstrating the most significant reduction (Fig. 3K). We next established IGF1R-overexpressing LD1 cell lines and performed orthotopic liver injections in C57BL/6 mice (Fig.S4I). Similarly, overexpression of IGF1R markedly exacerbated tumor growth, treatment with surufatinib significantly reduced tumor burden in oeIGF1R-expressing mice, an effect that was further enhanced by the addition of the IGF1R inhibitor linsitinib (Fig.S4J, K). Finally, to investigate the impact of IGF1R phosphorylation on the sensitivity to surufatinib treatment, we generated IGF1R mutants at residues Y1165/1166 and performed in vivo experiments. Compared with IGF1R overexpression alone, activation mutations at Y1165/1166 further diminished the inhibitory effect of surufatinib on tumor growth, whereas inactivation mutations at these sites reduced tumor burden and rendered tumors more responsive to surufatinib treatment (Fig. 3L–N), underscoring the critical role of IGF1R Y1165/1166 phosphorylation in determining surufatinib sensitivity.

Taken together, IGF1R is elevated in CCA tumors and predicts poor survival. Functionally, surufatinib-resistant cells and tissues display bypass activation of IGF1R—markedly increased p-IGF1R^Y1165/1166^ and PI3K–AKT/MEK–ERK signaling—despite unchanged total IGF1R. Genetic or pharmacologic dampening of IGF1R signaling restores drug response and, in vivo, surufatinib + linsitinib effectively restrains tumor growth; conversely, IGF1R overexpression or Y1165/1166-activating mutants blunt surufatinib efficacy. Collectively, these results establish IGF1R phosphorylation—especially at Y1165/1166—as a key determinant of TKI sensitivity and support IGF1R blockade as a rational partner for surufatinib in CCA.

### Structure basis of recognition and dephosphorylation of IGF1R^Y1165/1166^ by PTPN9

**Fig. 4:**
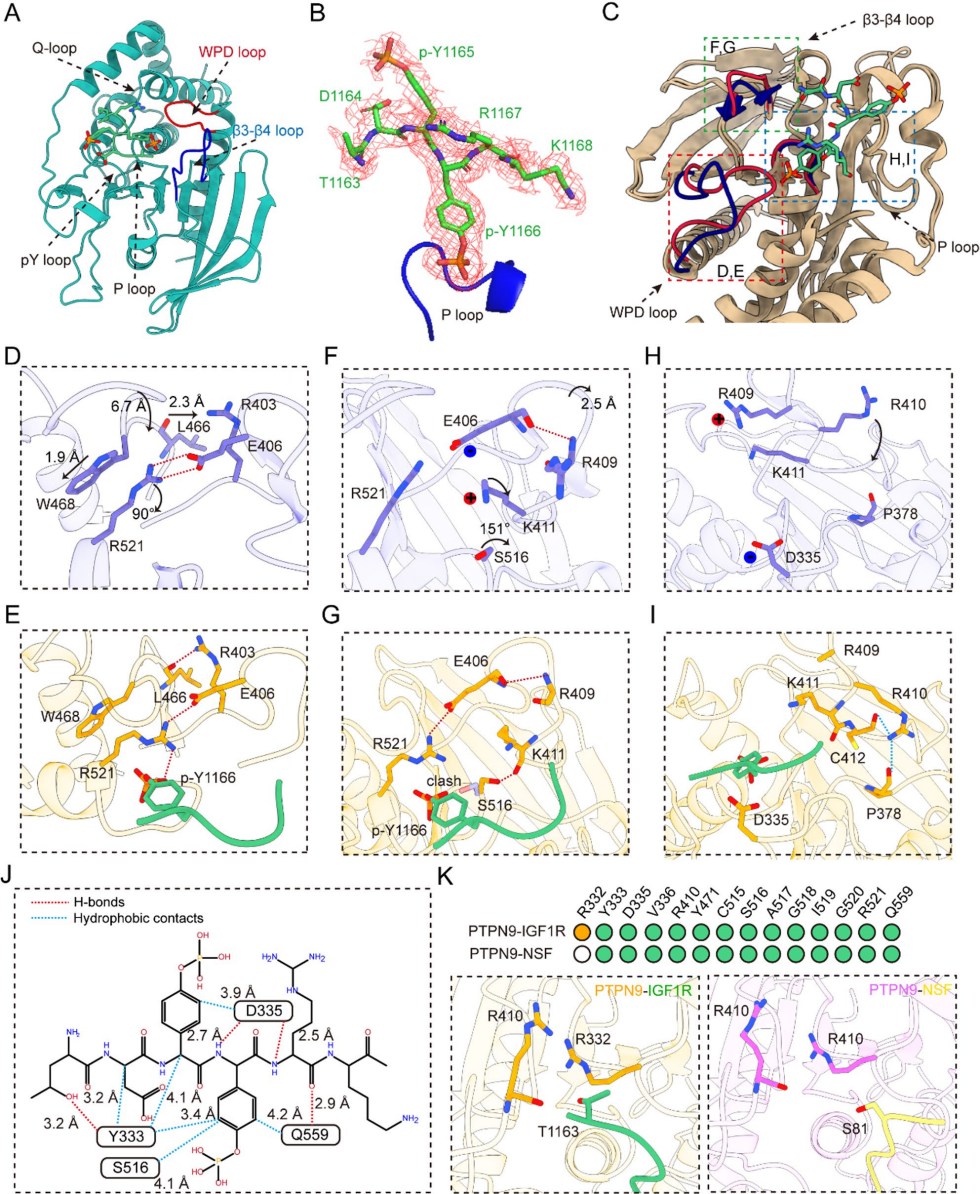
Structural insight of the molecular mechanism by which PTPN9 regulates the dephosphorylation of IGF1R at Y1165/Y1166. **A** The three-dimensional (3D) representation of PTPN9-C^515^S/D^470^A in complex with the p-IGF1R^Y1165/1166^ phospho-segment. **B** The 2Fo-Fc annealing omit map of p-IGF1R^Y1165/1166^ phospho-segment, the P-loop was colored in blue. **C** The three-dimensional (3D) representation of superposition of PTPN9-C^515^S/D^470^A-IGF1R complex and PTPN9 inactive protein structure (PDB: 2PA5, blue). The WPD loop, β3-β4 loop and P-loop of PTPN9-C^515^S/D^470^A-IGF1R complex or PTPN9 inactive protein structure were highlighted by red or blue, respectively. **D**-**E** Three-dimensional (3D) representation of detailed conformational changes of WPD-loop in PTPN9-C^515^S/D^470^A-IGF1R complex (**D**) and PTPN9 inactive protein structure (**E**). The rotation of R^521^ leads to the shift of W^468^ and a corresponding 6.7 Å movement of WPD-loop with a H-bond formed by L^466^ and R^473^. **F**-**G** Three-dimensional (3D) representation of detailed conformational changes of β3-β4 loop in PTPN9-C^515^S/D^470^A-IGF1R complex (**F**) and PTPN9 inactive protein structure (**G**). The disruption of the salt bridge between E^406^ and R^521^ and the rotation of S^516^ leads to the conformational changes of β3-β4 loop. **H**-**I** Three-dimensional (3D) representation of detailed conformational changes of P-loop in PTPN9-C^515^S/D^470^A-IGF1R complex **H** and PTPN9 inactive protein structure **I**. The rotation of R^410^ leads to the polar interactions between R^410^ and P^378^/C^412^ contributed to conformation change of P-loop. **J** Diagram of the p-IGF1R^Y1165/1166^ phospho-segment interaction in the active pocket of PTPN9-C^515^S/D^470^A-IGF1R complex. The blue or red dash was indicated the hydrogen bonds or hydrophobic interaction. **K** The difference interface pattern of different PTPN9 complex. (Top panel) Barcode representation of interaction pattern in the interface of PTPN9 bound by different substrate, p-IGF1R^Y1165/1166^ phospho-segment or p-NSF^Y83^ phospho-segment, respectively. (Bottom panel) Three-dimensional (3D) representation of detailed interactions difference in the interface between PTPN9-C^515^S/D^470^A-p-IGF1R^Y1165/1166^ complex and PTPN9-C^515^S/D^470^A-p-NSF^Y83^complex

To further understand how PTPN9 dephosphorylates IGF1R^Y1165/1166^, we obtained the electron density cloud of the complex of IGF1R^Y1165/1166^ short peptide and PTPN9 by X-diffraction crystallography with an overall resolution of 2.0 Å. After structural determination and refinement, the data of the final refined structure is shown in Supplemental Table 5. In the crystal diffraction junction of IGF1R^Y1165/1166^ peptide-PTPN9, the density of T^1163^DpYpYRK^1168^ of IGF1R^Y1165/1166^ short peptide extends into the catalytic center of PTPN9 (Fig. 4A, B). Further, we superimposed the IGF1R^Y1165/1166^ peptide-PTPN9 structure with the inactive-PTPN9 structure. Analysis by superposition revealed that the WPD loop of the PTPN9 underwent a significant inward shift, however the β3-β4 loop underwent an outward shift (Fig. 4C). In the WPD loop change, p-Y1166 of the IGF1R^Y1165/1166^ peptide reached into the catalytic pocket to form a hydrogen bond with R521 of PTPN9, causing W468 to move toward R521. the movement of W468 caused the WPD loop main chain to move closer to 6.7 Å inside the catalytic center and stabilize the WPD through the formation of a hydrogen bond between L466 and R403 loop conformation (Fig. 4D, E). In the β3-β4 loop, the S516 side chain is deflected by 151° due to the p-Y1166 reach-in and is hydrogen bonded to K411. This conformational change deflects the side chain of K411 and releases the charge interaction with E406, while causing the β3-β4 loop shifted outward by 2.5 Å (Fig. 4F, G). In addition, the β3-β4 loop of R410 in the β3-β4 loop was shifted outward by 2.5 Å (Fig. 4F, G). P loop was flipped and formed polar interactions with P378 and C412, further stabilizing the β3-β4 loop conformation (Fig. 4H, I). Subsequently, we analyzed the sites where the PTPN9 catalytic pocket interacts with the IGF1R^Y1165/1166^ short peptide and compared with the catalytic pocket of PTPN9-C515S/D470A-NSF-pY83 complex. The comparison showed both common and distinct interactions (Fig. 4J, K). In the pY core (p^Y1166^/p^Y83^), the peptide segments both facilitate hydrogen bonds with Y333, S516, I519, R521 and cation-π with Y471. Outside the pY core, the T1163 of IGF1R^Y1165/1166^ interacted R332 and main chain of R410 with hydrogen bond.

### PTPN9 exhibits substrate preference and site-specific phosphatase activity toward IGF1R^Y1165/1166^

**Fig. 5:**
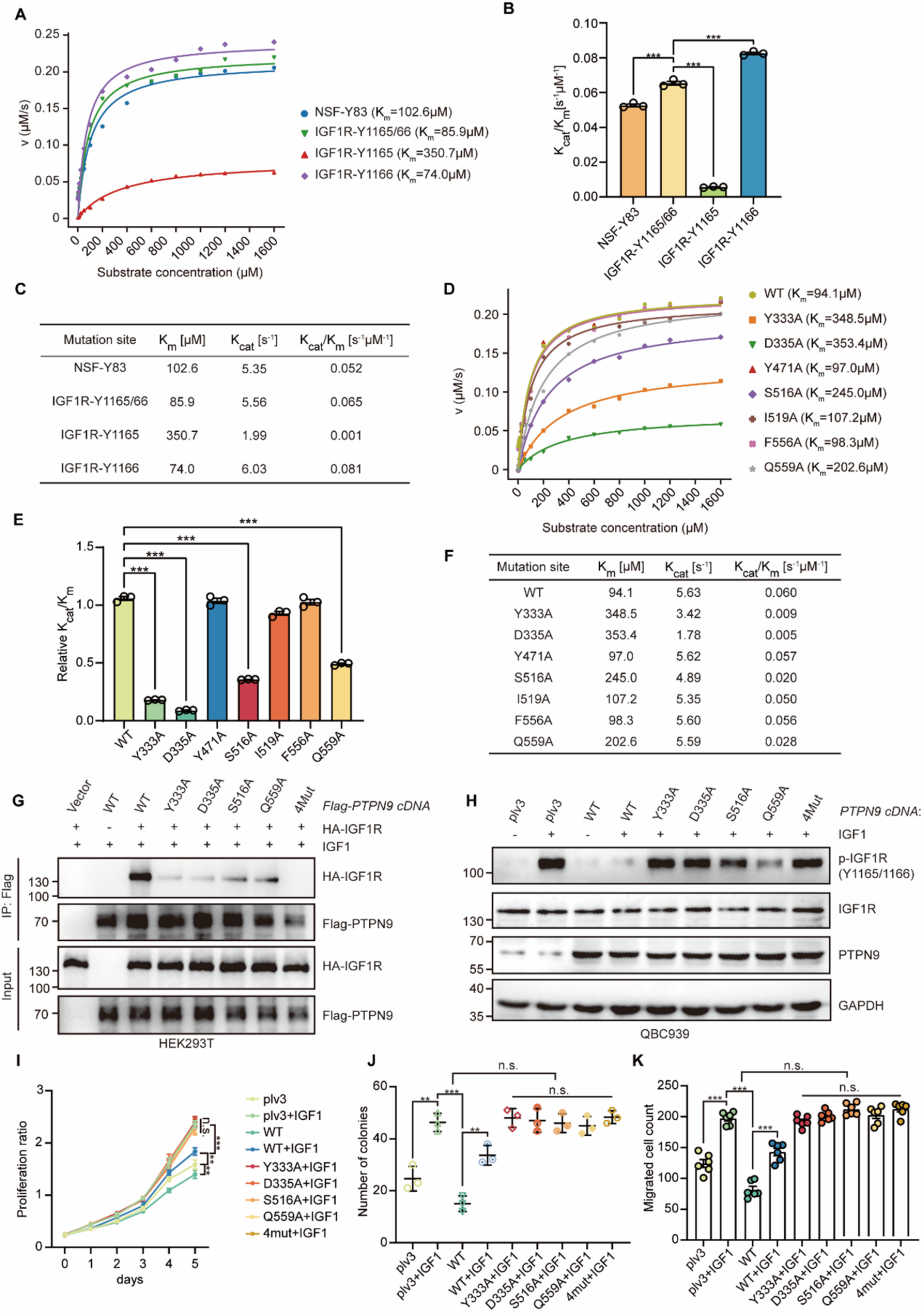
Y333/D335 is essential for PTPN9-IGF1R interaction and subsequent CCA progression.**A** Michaelis–Menten kinetic analysis of WT PTPN9 using different phospho-substrates—including IGF1R^Y1165/1166^, IGF1R^Y1165^, IGF1R^Y1166^, NSF^Y83^, and demonstrates substrate specificity. **B** Bar graph comparing catalytic efficiencies (K_cat_/K_m_) of WT PTPN9 on each phospho-substrate, showing that PTPN9 exhibits relatively lower affinity and enzymatic activity toward IGF1R^Y1165^. **C** Table summarizing K_m_, K_cat_, and K_cat_/K_m_ values for WT PTPN9 on all tested substrates. **D** Michaelis–Menten kinetic curves of WT and mutant PTPN9 proteins using a p-IGF1R^Y1165/1166^ peptide (0–1600 µM) as substrate. **E** Quantification of catalytic efficiency (K_cat_/K_m_) of WT and PTPN9 mutants; Y333A and D353A significantly reduce enzymatic activity. **F** Kinetic parameters (K_m_, K_cat_, and K_cat_/K_m_) for WT and mutant PTPN9 enzymes on IGF1R^Y1165/1166^, showing that mutations at Y333A and D353A substantially impair catalytic efficiency. **G** Co-immunoprecipitation analysis of the interaction between PTPN9 and IGF1R in HEK293T cells. Cells were transfected with HA-tagged IGF1R and either Flag-tagged WT PTPN9, PTPN9 point mutants (Y333A, D335A, S516A, Q559A), or a quadruple mutant (4Mut), followed by IGF1 stimulation. **H** Western blot analysis of p-IGF1R^Y1165/1166^, IGF1R, and PTPN9 levels in QBC939 cells transfected with PTPN9 (WT) or the indicated point mutants (Y333A, D335A, S516A, Q559A, or 4mut) and treated with or without IGF1 (10 ng/mL). GAPDH was used as a loading control. **I** Cell proliferation curves of indicated groups over 5 days. **J** Colony formation assay showing the number of colonies formed under each condition. **K** Transwell migration assay showing the number of migrated cells under each condition Data are from at least three independent experiments and are presented as mean ± SD. Statistical comparisons were performed using unpaired t-test (**B**, **E**, **I**-**K**). Significance indicators: *P < 0.05, **P < 0.01, ***P < 0.001

To assess substrate selectivity, we performed kinetic comparisons using five phosphotyrosine-containing peptides: p-IGF1R^Y1165/1166^, p-IGF1R^Y1165^, p-IGF1R^Y1166^, and p-NSF^Y83^. Wild-type PTPN9 exhibited high catalytic efficiency toward p-IGF1R^Y1165/1166^, and p-NSF^Y83^ peptides (Fig. 5A). Notably, PTPN9 exhibited low activity towards the peptide containing only the pY1165 site which is indicated by low K_cat_ and high K_m_, whereas the pY1166-only peptide yielded kinetic parameters slightly better than the dual-phosphorylation substrate (Fig. 5B, C), suggesting Y1166 as the preferable dephosphorylation substrate.

To further validate the key findings from the PTPN9-IGF1R crystal structure. A panel of seven PTPN9 mutants targeting conserved residues within its catalytic domain were generated (Fig. S5A), purified, and assessed for phosphatase activity against a p-IGF1R^Y1165/1166^ peptide (Fig. 5D). Among these, the Y333A mutant exhibited a K_m_ of 348.5 µM and a K_cat_ of 3.42 s^− 1^, whereas the D335A mutant showed a K_m_ of 353.4 µM and a K_cat_ of 1.78 s^− 1^, resulting in markedly reduced catalytic efficiencies (Fig. 5E). Correspondingly, the catalytic efficiencies (K_cat_/K_m_) of Y333A and D335A were substantially lower compared with the wild-type protein and other tested mutants (Fig. 5F). These results highlight the essential roles of Tyr333 and Asp335 in maintaining substrate binding and catalytic function, consistent with prior structural conclusions. Exogenous overexpression of HA-IGF1R and different Flag-PTPN9 mutant in HEK293T cells further confirmed that Tyr333, Asp335 are both key site in mediating the interaction between PTPN9 and IGF1R protein (Fig. 5G); To further confirm the function of Tyr333, Asp335 site for PTPN9, we established different stable overexpression of PTPN9 WT and mutant cell lines with QBC-939, only overexpression PTPN9 WT remarkedly decreased IGF1-stimulated IGF1R^Y1165/1166^ phosphorylation, Y333A and D335A failure to exhibit sufficient phosphatase activity of PTPN9 (Fig. 5H). Then, functional assays also showed the similar results (Fig. 5I-K, Fig. S5B).

Collectively, these biochemical data confirmed that IGF1R-Y1166 was a preferentially substrate of PTPN9, and established that the integrity of catalytic residues Tyr333 and Asp335 is crucial for the PTPN9-IGF1R interaction.

### CAF-derived IGF1 activates IGF1R and promotes CCA progression

**Fig. 6:**
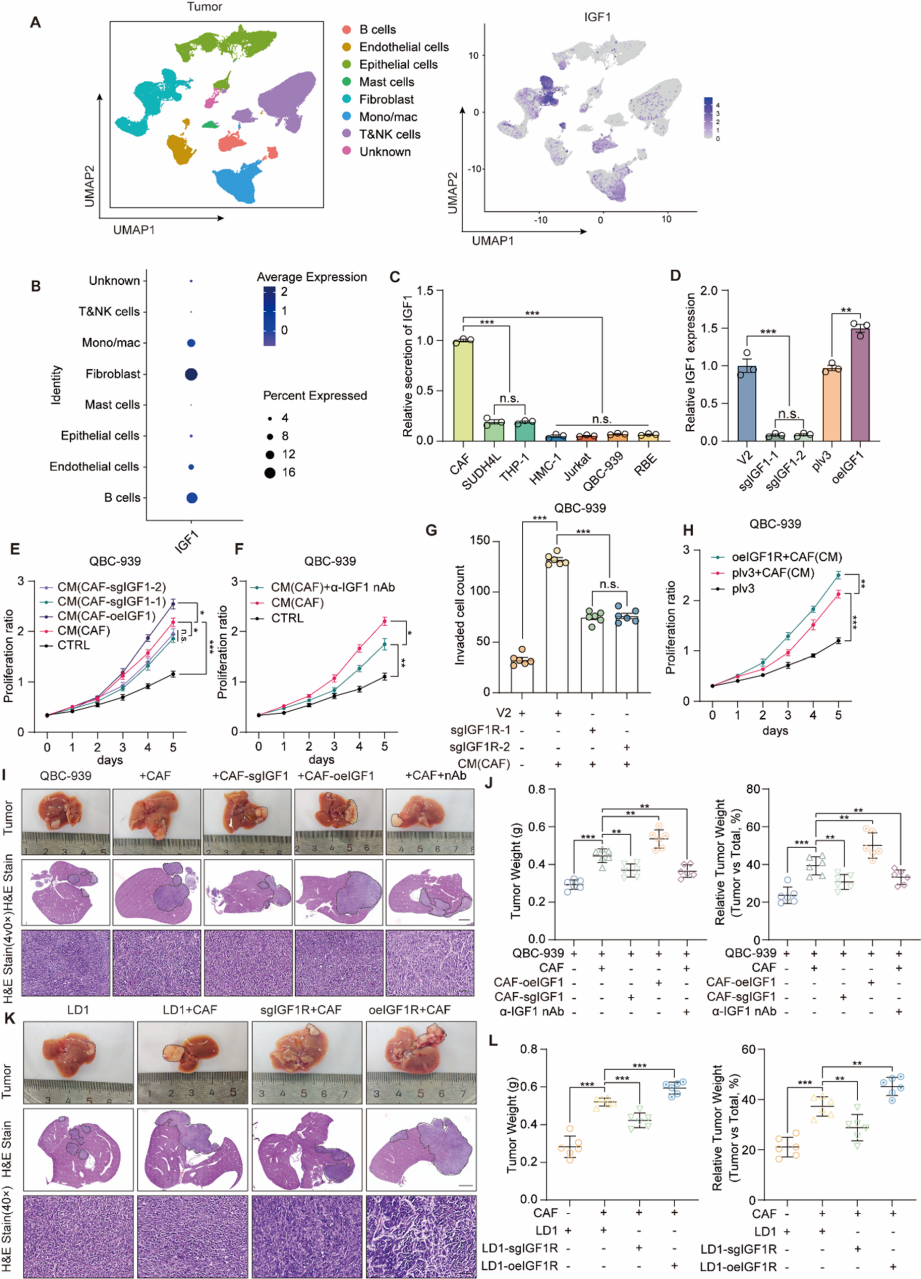
Cancer-associated fibroblast-derived IGF1 activates IGF1R signaling and drive cholangiocarcinoma growth and invasion.**A** Left: UMAP plot showing the distribution of cell populations. Right: IGF1 expression in different cell populations. **B** Dot plot showing the expression of IGF1 in different cell types. **C** ELISA showing IGF1 secretion levels in different cell types. **D** ELISA showing IGF1 secretion levels in CM from sgNC, sgIGF1-1/−2, plv3, and oeIGF1 CAFs. **E** Proliferation assay was performed in QBC‑939 cells treated with CM from CAFs, sgIGF1 CAFs, or oeIGF1 CAFs. **F** Proliferation assay was performed in QBC‑939 cells treated with CAF‑CM ± α-IGF1 nAb. **G** Transwell invasion assay showing that sgIGF1R decreases CAF‑CM‑induced invasiveness in QBC‑939 cells. **H** Proliferation assay showing that oeIGF1R enhances the effect of CAF‑CM on the proliferation ability of QBC‑939 cells. **I** Orthotopic liver CCA model in NOD/SCID mice (*n* = 6 per group) was built by injecting with QBC-939 cells alone, or co-injecting with CAFs, oeIGF1 CAFs, sgIGF1 CAFs, or treated with IGF1-neutralizing antibody. Tumor regions are outlined with dashed lines. Scale bars: 2 mm (middle panels), 25 μm (lower panels). **J** Tumor weight (left) and relative tumor weight (right) in the orthotopic model. **K** Orthotopic liver CCA model in C57BL/6 mice (*n* = 6 per group) was built by injecting with LD1 cells alone, or co-injected with CAFs, sgIGF1R + CAF, or oeIGF1R + CAF. Tumor regions are outlined with dashed lines. Scale bars: 2 mm (middle panels), 25 μm (lower panels). **L** Tumor weight (left) and relative tumor weight (right) in the orthotopic model Data are from at least three independent experiments and are presented as mean ± SD or representative images. Statistical analyses were performed using unpaired t-test (**C**, **D**, **G**, **J**, **L**) and two-way ANOVA (**E**, **F**, **H**). Significance indicators: n.s., not significant, *P < 0.05, **P < 0.01, ***P < 0.001

To identify the source of IGF1 responsible for IGF1R activation in vivo, we analyzed single-cell RNA sequencing data and observed predominant IGF1 expression in fibroblasts (Fig. 6A, B). Subsequently, we isolated CAFs from freshly resected tumor tissues (Fig. S6A, B) and selected representative cell lines corresponding to the major cell clusters identified in the single-cell dataset. Consistent with the single-cell RNA sequencing data, IGF1 was found to be relatively highly expressed in fibroblasts based on qPCR assay of multiple cell types (Fig. S6C). To further confirm these findings, we collected conditioned media (CM) from these cell lines and measured IGF1 secretion using ELISA kit, demonstrating significantly higher IGF1 secretion by CAFs compared to other cell types (Fig. 6C). Notably, THP-1 and SU-DHL-4 cells also exhibited relatively high levels of IGF1 expression and secretion. To determine whether CAF-derived cytokines predominantly contribute to the tumor-promoting effects, we collected CM from all three cell lines and co-cultured them with tumor cells. The results revealed that CAF-CM, rather than CM from THP-1 or SU-DHL-4, more potently enhanced tumor cell proliferation, migration, and invasion (Fig. S6D–G), underscoring the dominant role of CAFs in promoting tumor progression. We then generated CAF-sgIGF1 clones and CAF-oeIGF1 cell lines, verified by Western blotting assay (Fig. S7H). ELISA analysis of CM from sgNC, sgIGF1-1, sgIGF1-2, plv3, oeIGF1 cells showed significantly increased IGF1 secretion in oeIGF1 CAF cells and significantly decreased secretion in sgIGF1 cells compared to controls (Fig. 6D). Function assays showed that CM from CAF cells significantly promoted tumor cell proliferation compared to control medium, and this promoting effect was enhanced with CM from CAF-oeIGF1 and reduced using CM from sgIGF1 cells (Fig. 6E). Migration and invasion assays demonstrated similar patterns (Fig. S7A, B).

To further verify that CAF-derived IGF1 promotes tumor progression, we introduced anti-IGF1 neutralizing antibody (α-IGF1 nAb) into CM from CAF cells and performed functional assays. The addition of α-IGF1 nAb significantly diminished CAF-CM-mediated enhancement of tumor cell proliferation (Fig. 6F), colony formation (Fig. S7C) and migration (Fig. S7D). To confirm IGF1’s action through IGF1R, we conducted experiments using sgIGF1R QBC-939 and RBE cells. Transwell assays demonstrated significantly reduced migration and invasion in sgIGF1R cells treated with CM from CAF compared to controls also treated with CM from CAF (Fig. 6G, Fig. S7E). Colony formation (Fig. S7F) yielded similar results. Conversely, oeIGF1R cells exhibited enhanced proliferation in response to CM(CAF) compared to vector controls, with consistent findings in migration assay (Fig. 6H, Fig. S7G, H). Finally, using PTPN9-overexpressing cholangiocarcinoma cells cultured in CM from CAF, we observed that PTPN9 overexpression partially mitigated the enhanced migration and invasion induced by CM(CAF) compared to vector controls (Fig. S7I, J).

An orthotopic mouse liver tumor model was established in NOD/SCID mice to further evaluate the role of CAF-derived IGF1 in promoting CCA progression in vivo. Tumor cells were implanted alone or co-injected with CAFs, CAF-oeIGF1, or CAF-sgIGF1 cells, with or without IGF1-neutralizing antibody. Co-injection with CAFs markedly increased tumor burden compared to tumor cells alone, as evidenced by gross liver morphology and H&E staining. Tumor growth was further enhanced in the CAF-oeIGF1 group, while both CAF-sgIGF1 and CAF + α-IGF1 nAb groups showed significantly reduced tumor sizes compared to CAF + oeIGF1 group (Fig. 6I, J), indicating a tumor-promoting function of CAF-derived IGF1. To determine whether this effect is mediated through tumor cell IGF1R, LD1 cells with IGF1R knockout or overexpression were orthotopically injected along with CAFs (Fig. S7K). Tumors formed by sgIGF1R cells were significantly smaller compared to WT LD1, whereas those derived from oeIGF1R cells exhibited greater tumor burden compared to controls (Fig. 6K, L). These findings reinforce the critical role of the CAF–IGF1–IGF1R axis in driving cholangiocarcinoma progression in vivo. To further elucidate the role of CAFs in surufatinib resistance, we conducted functional assays and found that the inhibitory effects of surufatinib on tumor cell phenotypes were markedly attenuated in the presence of CAF-CM (Fig. S8A–D), suggesting that CAFs may contribute to surufatinib resistance.

Building on these findings, we revisited the scRNA-seq data to assess IGF1 expression in CAFs from iCCA, pCCA, and dCCA and thereby infer the predominant subtype. The results showed that IGF1 expression was consistently higher in CAFs among all three subtypes (Fig. S8E, F). We then isolated CAFs from the three types of CCA tissues and measured IGF1 secretion using ELISA, which revealed that all CCA-derived CAFs secreted markedly higher levels of IGF1 than other cell types, with pCCA-CAFs exhibiting the highest secretion levels (Fig. S8G). Moreover, CAFs secreted significantly more IGF1 than normal fibroblasts (NFs) (Fig. S8H). Subsequently, conditioned media (CM) from iCCA-, pCCA-, and dCCA-derived CAFs were collected and used to co-culture with tumor cells. The results demonstrated that pCCA-CAF–derived CM more strongly promoted tumor cell proliferation, migration, and invasion compared with CM from iCCA- or dCCA-CAFs, while no significant differences were observed between the latter two groups (Fig. S8I–L), indicating that pCCA-CAFs may play a more dominant role in promoting tumor progression.

Collectively, these data identify CAFs as the principal source of IGF1 in the CCA microenvironment and demonstrate that CAF-derived IGF1 activates tumor-cell IGF1R to drive proliferation, migration, invasion, and tumor growth. These effects are strengthened by IGF1 overexpression and blunted by IGF1 knockdown or neutralization in CAFs, and are reduced by tumor-cell IGF1R loss but enhanced by IGF1R gain; PTPN9 overexpression partially counteracts CAF-CM–induced motility. CAF-CM also attenuates the anti-tumor activity of surufatinib, indicating that CAFs play an important role in the development of surufatinib resistance. Subtype analyses further show that pCCA-derived CAFs secrete the highest levels of IGF1 and exert the strongest pro-tumorigenic effects, highlighting the CAF–IGF1–IGF1R axis—particularly in pCCA—as a tractable therapeutic vulnerability.

## Discussion

The present study identifies a CAF-derived IGF1/IGF1R axis-constrained physiologically by the phosphatase PTPN9-as a central driver of CCA growth and, critically, of resistance to the multi-target TKI surufatinib. By integrating multi-omic profiling, functional assays and in vivo modelling, we extend current knowledge on bypass signaling in biliary tract tumors and offer a biomarker-guided combination strategy to restore drug sensitivity.

IGF1R emerged from our co-IP coupled LC-MS/MS screen and validated as a over-expressed receptor tyrosine kinase across TCGA-CHOL and our independent cohort, and its high expression correlated with shortened overall survival. These data align with consensus that place IGF-axis activation among the oncogenic hallmarks of CCA [2]. Functionally, forced IGF1R activation in QBC-939 and RBE cells enhanced proliferation, invasion and orthotopic tumor growth, whereas pharmacologic or genetic blockade produced the opposite phenotype, confirming a causal role for IGF1R in disease aggressiveness. Despite modest clinical outcomes from prior trials of IGF1R inhibitors in other cancers [31], the robust association between IGF1R expression and patient survival in CCA suggests that selecting patients based on IGF1R status could effectively enrich for therapeutic responders. Our work is the first to comprehensively validate IGF1R upregulation across all CCA subtypes and link it to poor prognosis, underscoring the clinical significance of the IGF axis in biliary tract cancers.

Tumor-stroma single-cell transcriptomics pinpointed cancer-associated fibroblasts (CAFs) as the dominant source of IGF1. Our observation that conditioned medium from IGF1-over-expressing CAFs robustly stimulated malignant phenotypes in CCA cells is consistent with the CAF heterogeneity map, which highlights growth factor-enriched iCAF subsets as key tumor promotors in intrahepatic CCA [32]. Importantly, neutralisation of secreted IGF1 or genetic ablation of IGF1R in tumor cells abrogated these effects, substantiating an essential paracrine loop. This finding adds to the growing body of literature recognizing that desmoplastic stroma in CCA is not merely a structural component but actively drives tumor progression [33]. Likewise, fibroblast-derived IGF1 can induce epithelial–mesenchymal transition in cancer cells, contributing to therapy resistance [34].

PTPN9 filled an unexplored niche in this circuitry: proteomic pull-down and structural modelling demonstrated a direct interaction with the IGF1R activation loop, and kinetic assays identified Asp335 as a catalytic hot spot governing Y1166 dephosphorylation. While phosphatases such as PTP1B and SHP2 are recognized modulators of RTK output [35–37], evidence for PTPN9 as a tumor suppressor has been largely restricted to metabolic contexts [38]. Our data therefore position PTPN9 loss as a previously unappreciated mechanism that unleashes IGF1R signaling in CCA.

Surufatinib, an oral inhibitor of VEGFR1-3, FGFR1 and CSF1R [39, 40], has shown durable activity in neuroendocrine tumors [41]. By stepwise drug escalation we generated the QBC-939/SR sub-line, revealing hyper-phosphorylation of IGF1R, Akt and Erk despite potent inhibition of VEGFR targets. Clinicopathological analysis of patient biopsies at radiologic progression corroborated this pattern, indicating that IGF1R re-engagement is a clinically relevant escape route. Similar bypass rewiring via IGF1R has been described after prolonged EGFR inhibition in CCA [42] and after FGFR blockade in fusion-positive models [43, 44], suggesting convergent adaptive trajectories irrespective of the primary TKI.

Notably, PTPN9 has been implicated across several RTK axes that are clinically targeted by TKIs. In breast cancer cells, PTPN9 directly dephosphorylates EGFR and ErbB2, attenuating downstream STAT3/STAT5 signaling, suggesting that PTPN9 status could influence responses to EGFR-directed TKIs [45, 46]. During anti-HER2 therapy, PTPN9 regulates HER3 phosphorylation, and loss of PTPN9 has been associated with trastuzumab resistance, highlighting broader ErbB crosstalk [47]. In endothelial cells, PTPN9 dephosphorylates VEGFR2 at Tyr1175 and constrains VEGF signaling, providing a mechanistic link to the activity of VEGFR-targeting TKIs (e.g., sunitinib/sorafenib) [48]. Importantly for cholangiocarcinoma, PTPN9 has been shown to dephosphorylate FGFR2 at the activation loop (pY656/657) and to synergize with the FGFR inhibitor pemigatinib, supporting a role for PTPN9 in modulating FGFR-TKI efficacy [30]. Finally, EGFR inhibition can downregulate PTPN9 with compensatory activation of STAT3; combined EGFR and STAT3 blockade (e.g., erlotinib plus niclosamide) overcomes resistance in preclinical models, further pointing to a PTPN9–STAT3 node that may generalize across TKI contexts [49].

Therapeutically, we demonstrate that the dual IR/IGF1R inhibitor linsitinib restored TKI sensitivity in vitro and synergized with surufatinib to reduce tumor burden and Ki-67 indices in an orthotopic model. Early-phase trials have already established manageable safety profiles for linsitinib in solid tumors [50], supporting clinical translatability of this combination. Notably, re-expression of PTPN9 attenuated IGF1R phosphorylation and blunted the invasive response to CAF-derived IGF1 or surufatinib exposure, underscoring PTPN9 as a potential predictive biomarker and therapeutic lever.

Collectively, our findings refine the mechanistic framework of TKI resistance in CCA. Rather than isolated “on-target” mutations, our data highlight stromal ligand supply and phosphatase imbalance as parallel forces that reactivate survival pathways downstream of alternative RTKs. This paradigm parallels resistance phenomena in other biliary targets such as FGFR2, where selective inhibitors are thwarted by feedback EGFR signaling or gatekeeper mutations [43]. Conceptually, upfront or adaptive co-inhibition of the IGF1R node may therefore forestall multiple bypass tracks simultaneously, an approach already gaining traction in other solid tumors.

The clinical implications of our findings support a more personalized and proactive approach to cholangiocarcinoma therapy. Immunohistochemical evaluation of IGF1R phosphorylation and PTPN9 expression in baseline tumor biopsies may serve as a valuable tool to stratify patients who are most likely to benefit from IGF1R-directed combination therapies, thereby facilitating biomarker-driven clinical trial designs. Considering the manageable toxicity profile of linsitinib and the favorable tolerability of surufatinib in biliary tract cancers, early-phase combination studies are warranted. These studies could further explore the therapeutic impact of concurrently targeting additional stromal-derived signals, such as HGF or IL-6, to enhance anti-tumor responses. Furthermore, monitoring circulating tumor DNA for signs of emerging IGF1R pathway activation, along with serial plasma measurements of stromal IGF1, offers a promising non-invasive strategy for tracking disease progression. This could enable timely therapeutic escalation to counteract acquired resistance and prolong clinical benefit.

This study has several limitations that should be acknowledged. The investigation relied on a single drug-resistant subline, which may limit the diversity of resistance mechanisms captured. Although linsitinib effectively inhibited IGF1R in our models, its concurrent activity against the insulin receptor (IR) introduces the risk of metabolic side effects that could complicate clinical translation. To address this, future efforts could explore the use of next-generation IGF1R-selective degraders, which may offer enhanced target specificity and a more favorable safety profile.

**Fig. 7:**
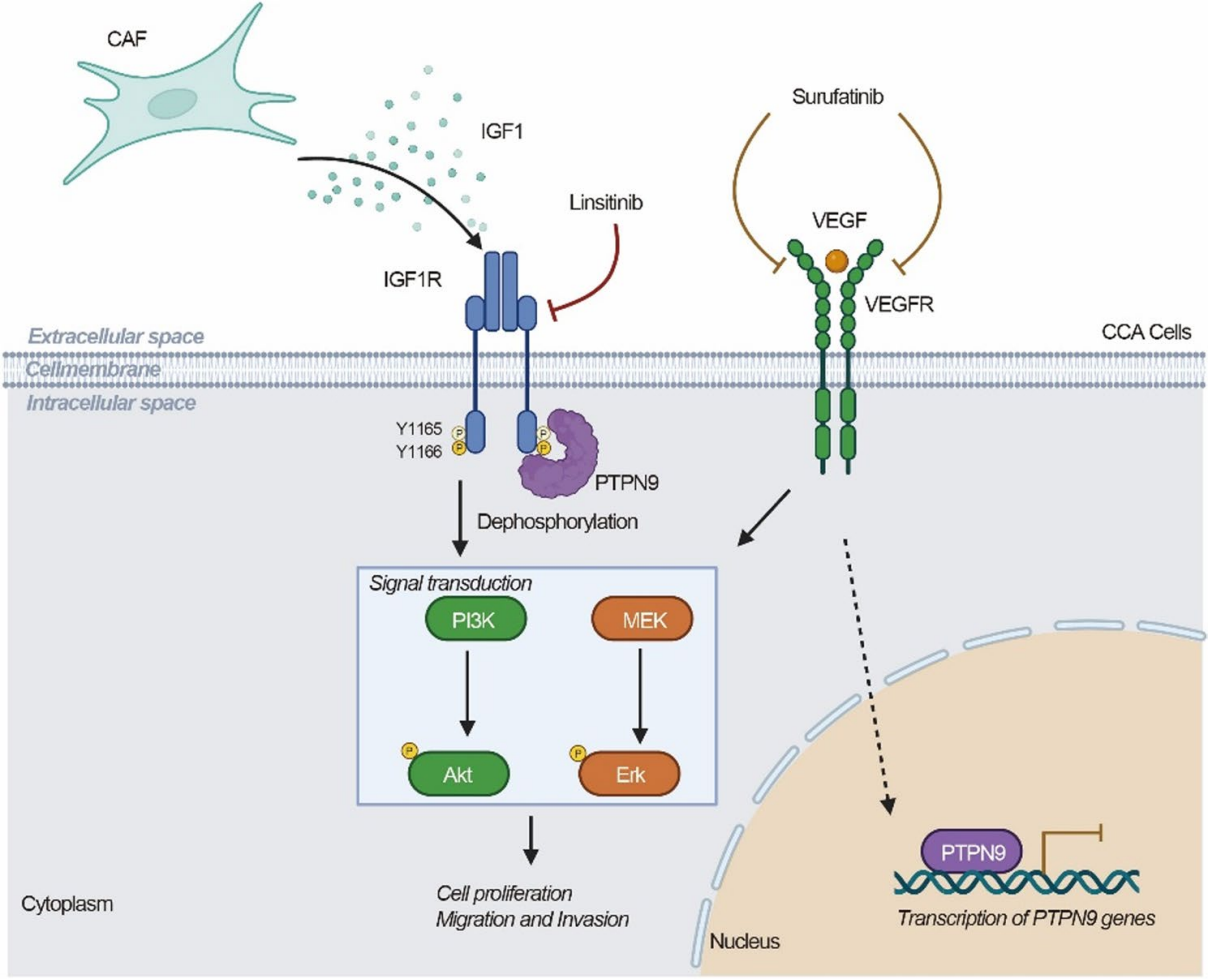
Schematic illustration of the CAF-derived IGF1/PTPN9–IGF1R signaling axis and its role in cholangiocarcinoma (CCA) progression and therapeutic resistance. Cancer-associated fibroblasts (CAFs) in the tumor microenvironment secrete high levels of IGF1, which activates IGF1R on CCA cells, leading to phosphorylation of Y1165/1166 and stimulation of downstream PI3K/Akt and MEK/Erk pathways, thereby promoting cell proliferation, migration, and invasion. The protein tyrosine phosphatase PTPN9 directly interacts with IGF1R and specifically dephosphorylates Y1165/1166, functioning as a negative regulator of IGF1R signaling. surufatinib targets VEGFR to inhibit tumor growth, and preliminary observations suggest that VEGFR signaling may also modulate PTPN9 expression. However, bypass activation of IGF1R contributes to therapeutic resistance. Downregulation of PTPN9 further enhances IGF1R-driven oncogenic signaling and confers resistance to surufatinib. Co-targeting IGF1R with linsitinib restores TKI sensitivity

In summary, our study delineates a previously underexplored CAF-derived IGF1/PTPN9-IGF1R signaling axis as a critical driver of cholangiocarcinoma progression and a key mediator of resistance to surufatinib. We demonstrate that loss of the phosphatase PTPN9 unleashes unchecked IGF1R signaling, fueling malignant growth and diminishing the therapeutic efficacy of tyrosine kinase inhibition. Importantly, our findings highlight the therapeutic value of dual targeting this axis-combining the IGF1R inhibitor linsitinib with surufatinib significantly enhances anti-tumor effects in preclinical models, underscoring a synergistic strategy to overcome resistance and achieve deeper, more sustained responses (Fig. 7). This combinatorial approach not only addresses the limitations of monotherapy but also exploits a vulnerability within the tumor-stroma crosstalk. Moving forward, biomarker-guided clinical trials integrating both tumor-intrinsic and microenvironmental cues-particularly IGF1R phosphorylation status and PTPN9 expression-will be instrumental in refining patient selection and optimizing therapeutic benefit. Collectively, these findings provide a compelling rationale for clinical evaluation of linsitinib and surufatinib co-administration as a precision-based strategy to improve outcomes in biliary tract cancers.

## Supplementary Information

Supplementary Material 1.

## Data Availability

All data generated or analyzed during this study are included in this article and its supplementary material files. Further inquiries can be directed to the corresponding author.

## Abbreviations

IGF1R: Insulin-like growth factor 1 receptor
PTPN9: Protein tyrosine phosphatase, non-receptor type 9
VEGFR: Vascular endothelial growth factor receptor
IR: Insulin receptor
HSP90: Heat shock protein 90
NSF: N-ethylmaleimide-sensitive factor
RTK: Receptor tyrosine kinase
TKI: Tyrosine kinase inhibitor
mTKI: Multi-target tyrosine kinase inhibitor
PTK: Protein tyrosine kinase
PTP: Protein tyrosine phosphatases
AAs: Amino acids
WT: Wild type
KO: Knockout
OE: Over-expression
CCA: Cholangiocarcinoma
iCCA: Intrahepatic CCA
pCCA: Perihilar CCA
dCCA: Distal CCA
PR: Partial response
PD: Progressive disease
SR: Surufatinib-resistant
Suru: Surufatinib
Lins: Linsitinib
Comb: Combination
DMSO: Dimethyl sulfoxide
CAF: Cancer-associated fibroblast
NF: Normal fibroblast
CM: Conditioned media
ELISA: Enzyme-linked immunosorbent assay
H&E: Hematoxylin and eosin
UMAP: Uniform Manifold Approximation and Projection
TCGA-CHOL: The Cancer Genome Atlas cholangiocarcinoma
CT: Computed tomography
MRI: Magnetic resonance imaging
WT: Wild-type
IC50: Half-maximal inhibitory concentration
K_m_: Michaelis constant
K_cat_: Catalytic turnover number
ANOVA: Analysis of variance
CI: Confidence interval
HR: Hazard ratio
SD: Standard deviation
i.p.: Intraperitoneal injection
i.g.: Intragastric administration
mut: Mutations
nAb: Neutralizing antibody
