# Supplementary material for: PTPN9 dephosphorylates IGF1RY1165/1166 and alleviates IGF1R-mediated resistance to tyrosine kinase inhibitor in cholangiocarcinoma
Source: J Exp Clin Cancer Res. 2025 Nov 22;44:332. doi: 10.1186/s13046-025-03594-2 (PMC12751603; doi:10.1186/s13046-025-03594-2)
Supplement: Supplementary file 1 — Supplementary Material 1. [file 13046_2025_3594_MOESM1_ESM.docx]

**Supplemental Material for:**

**PTPN9 dephosphorylates IGF1R^Y1165/1166^ and alleviates IGF1R-mediated resistance to tyrosine kinase inhibitor in cholangiocarcinoma**

Jia-ming Hu^1,#^, Hui-qiang Liu^1,#^, Ming-hui Zhang^1,#^, Tian-li Chen^1^, An-da Shi^1^, Qiang Gao^1^, Yun-jia Liu^1^, Xin Wang^1^, Kai-yang Sun^1^, Jian Deng^1^, Yun-fei Xu^1^, Chang Pan^2,3,4*^, Kang-shuai Li^1,*^, and Zong-li Zhang^1,*^

^1^Department of General Surgery, Qilu Hospital, Cheeloo College of Medicine, Shandong University, Jinan, Shandong, 250012, P.R. China

^2^Department of Emergency Medicine, Qilu Hospital of Shandong University, 107 Wenhua Xi Road, Jinan 250012, China.

^3^Shandong Provincial Clinical Research Center for Emergency and Critical Care Medicine, Institute of Emergency and Critical Care Medicine of Shandong University, Chest Pain Center, Qilu Hospital of Shandong University, 107 Wenhua Xi Road, Jinan 250012, China.

^4^Key Laboratory of Emergency and Critical Care Medicine of Shandong Province, Key Laboratory of Cardiopulmonary-Cerebral Resuscitation Research of Shandong Province, Qilu Hospital of Shandong University, 107 Wenhua Xi Road, Jinan 250012, China.

#Jia-ming Hu, Hui-qiang Liu and Ming-hui Zhang contributed equally to this work.

**Methods and materials**

**Study cohorts**

This study involves five clinical cohorts. Cohort 1 (CCA cohort, Supplemental Table 6) consisted of a total of 427 primary CCA patients who underwent tumor resection at Qilu Hospital of Shandong University from 2010 to 2020. Tumors were categorized and staged according to the 8th edition of the AJCC/UICC TNM classification system, and the patient prognosis was monitored through follow-up. All enrolled CCA patients had not received any adjuvant therapy prior to inclusion. Cohort 2 (CCA vs. normal tissue cohort, Supplemental Table 7) consisted of a total of 81 primary CCA patients who underwent tumor resection at Qilu Hospital of Shandong University from 2010 to 2020. Paired tumor and para-tumor normal tissues were collected to analyze the differential expression between tumor and normal tissues. Cohort 3 (Surufatinib-treatment cohort, Supplemental Table 8) consisted of 24 patients in unresectable and advanced stages who have received surufatinib treatment in Qilu Hospital of Shandong University from September 2022 to September 2024. These patients were from a prospective, single-arm, open-label, multi-center, observational real-world clinical study to observe and evaluate the efficacy and safety of surufatinib in the treatment of patients with biliary tract cancer (BTC). The key eligibility criteria were Age ≥18, male or female; histologically or cytologically confirmed unresectable or metastatic BTC, including intrahepatic cholangiocarcinoma (iCCA), extrahepatic cholangiocarcinoma (eCCA), and gallbladder cancer (GBC); ECOG score 0-2; Confirmed measurable (or evaluable) lesions that meet the requirements of RECIST 1.1. Eligible participants received oral surufatinib (300 mg) once daily. Tumor imaging assessments are performed every 8 weeks via CT or MRI. The Response Evaluation Criteria in Solid Tumors (RECIST 1.1) are used to evaluate objective tumor response. According to RECIST 1.1, efficacy is categorized as complete response (CR; disappearance of all target lesions with no new lesions, sustained for ≥4 weeks), partial response (PR; ≥30% decrease in the sum of diameters of target lesions from baseline, sustained for ≥4 weeks), stable disease (SD; a decrease that does not meet PR or an increase that does not meet PD), and progressive disease (PD; ≥20% increase in the sum of diameters of target lesions compared with the nadir and an absolute increase of ≥5 mm, or the appearance of new lesions, or unequivocal progression of non-target lesions). Cohort 4 (CAF isolation cohort, Supplemental Table 9) consisted of 12 intrahepatic CCA patients, 15 perihilar CCA patients and 3 distal CCA patients who underwent tumor resection from May 2024 to July 2025. Fresh tumor tissues were collected for isolation of CAF. IGF1/PTPN9 co-localization immunofluorescence cohort (Cohort 5) was obtained from needle biopsy tissues of 3 response and 3 non-response patients in Cohort 3. These specimens were collected for immunofluorescence colocalization studies.

**Cells and Reagents**

The human intrahepatic cholangiocarcinoma (iCCA) line RBE (RRID: CVCL_4896), the human perihilar cholangiocarcinoma (pCCA) line QBC-939 (RRID: CVCL_6942), the human embryonic kidney line HEK293T (RRID: CVCL_0063), the murine cholangiocarcinoma line LD1 (RRID: CVCL_C7SL), the mast cell line HMC-1 (RRID: CVCL_0003), the monocyte line THP-1 (RRID: CVCL_0006), the T-cell line Jurkat (RRID: CVCL_0065), and the B-cell lymphoma line SU-DHL-4 (RRID: CVCL_0539) were obtained from the Cell Bank of the Chinese Academy of Sciences (Shanghai, China) between 2021 and 2024. All lines were authenticated by short tandem repeat (STR) profiling and confirmed to be mycoplasma-free. Cells were cultured in RPMI-1640 medium (Gibco) supplemented with 10% fetal bovine serum (Gibco) and 1% penicillin/streptomycin (Solarbio, P1400); HMC-1, THP-1, Jurkat, and SU-DHL-4 cells were further supplemented with 0.05 mM β-mercaptoethanol. All cultures were maintained at 37 °C in a humidified atmosphere containing 5% CO₂.

**Primary Isolation of CAFs From Human Cholangiocarcinoma Specimens**

Fresh intrahepatic or perihilar cholangiocarcinoma (CCA) specimens were obtained immediately after surgical resection under protocols approved by the Institutional Review Board of Qilu Hospital of Shandong University, with written informed consent from all patients. All procedures complied with the Declaration of Helsinki. Tumor tissue was transferred on ice to the laboratory in Dulbecco’s phosphate-buffered saline (DPBS) supplemented with 1% penicillin/streptomycin (P/S). Necrotic areas and visible bile ducts were removed with sterile scalpels. The sample was minced into ~1 mm³ fragments in a biosafety cabinet. Minced tissue was incubated at 37 °C for 45 min in digestion buffer (DMEM/F12 (Gibco, 11320033) containing 0.1% collagenase type I(MCE, HY-E70005A), 0.1% collagenase type IV (MCE, HY-E70005D), 0.01% hyaluronidase (MCE, HY-108903), and 80 U mL⁻¹ DNase I(MCE, HY-108882)) with gentle agitation (120 rpm). Every 15 min, the suspension was pipetted gently with a wide-bore tip to enhance dissociation. The digest was filtered sequentially through 100 µm (Beyotime, FSTR100) and 70 µm cell strainers (Beyotime, FSTR070) into ice-cold DMEM/F12 + 10% FBS. Cells were pelleted at 300 × g for 5 min, resuspended in growth medium (DMEM/F12, 10% FBS, 1% P/S, 1% non-essential amino acids, and 1 mM sodium pyruvate (Gibco, 11360070)), and seeded onto T-25 flasks pre-coated with 0.1% gelatin (MCE, HY-Y1365). After 2 h at 37 °C/5% CO₂, non-adherent tumor and immune cells were removed by gently rinsing twice with DPBS. Adherent cells were cultured in fresh growth medium, which was replaced every 48 h to eliminate residual epithelial cells. When cultures reached 70% confluence, cells were passaged 1:3 with 0.05% trypsin/EDTA. Early-passage cells were confirmed as CAFs by immunofluorescence (α-SMA (Cell Signaling Technology, 19245), FAP (Invitrogen, PA5-99458), and absence of epithelial markers (E-cadherin, pan-cytokeratin). Flow cytometry (BD FACSAria) further excluded EpCAM⁺ tumor cells (<1%) and CD45⁺ leukocytes (<2%). Validated CAFs were cryopreserved in 90% FBS/10% DMSO and stored in liquid nitrogen. Experiments were conducted with cells between passages 3 and 8 to maintain primary characteristics, and all cells were confirmed to be mycoplasma-free. CAFs or its culture medium (CM) from iCCA were used in the treatment of RBE cells and CAFs or its CM from pCCA were used in the treatment of QBC939 cells.

**Plasmid construction**

pcDNA3.1 plasmids encoding 3×Flag-tagged human PTPN9 and HA-tagged IGF1R were purchased from Miaolingbio (Wuhan, China). Overexpression constructs for human IGF1 (pLV3-CMV-IGF1), human PTPN9 (pLV3-CMV-PTPN9), and human IGF1R (pLV3-CMV-IGF1R) were also obtained from Miaolingbio. Point mutations of PTPN9 (Y333A, D335A, Y471A, S516A, I519A, F556A, and Q559A) and IGF1R (Y1165/66E, Y1165/66F) were introduced using the QuickChange Site-Directed Mutagenesis Kit (Agilent, USA). Overexpression plasmids for murine PTPN9 (pLV3-CMV-PTPN9) and IGF1R (pLV3-CMV-IGF1R) were likewise synthesized by Miaolingbio. All constructs were verified by Sanger sequencing before use.

**CRISPR-Cas9–Mediated Gene Knockout**

To knockout human or murine IGF1, IGF1R, and PTPN9, gene-specific sgRNAs (sgIGF1, sgIGF1R, sgPTPN9) were designed and cloned into the lentiCRISPR v2 vector (Addgene, 52961). A non-targeting sgRNA was used as the negative control. Lentiviral particles were produced by co-transfecting lentiCRISPR v2-sgRNA plasmids with psPAX2 and pMD2.G into HEK293T cells using Lipofectamine 3000. Viral supernatants were harvested after 48 h, filtered, and used to infect target cells in the presence of 8 μg/mL polybrene. sgRNA cell pools strategy were applied. After 24 hours of infection, cells were selected with Puromycin for at least 48 hours. A subset of cells was collected to confirm efficient target gene knockout by Western blotting, and the remaining cells were subjected to functional assays.

**Gene Overexpression and Knockout**

For transient expression experiments, HEK293T cells were transfected with pcDNA3.1-based plasmids encoding 3×Flag-PTPN9 or HA-IGF1R using Lipofectamine 3000 (Thermo Fisher Scientific) according to the manufacturer’s instructions. For stable overexpression, lentiviral particles were generated by co-transfecting pLV3-based constructs (encoding human or murine IGF1, IGF1R, or PTPN9) with psPAX2 and pMD2.G packaging plasmids into HEK293T cells. Supernatants were harvested 48 h post-transfection, filtered through a 0.45 μm membrane, and used to infect target cells in the presence of 8 μg/mL polybrene. Infected cells were selected with puromycin (2 μg/mL) for 3–5 days prior to downstream assays.

Gene knockout was achieved using the CRISPR-Cas9 system as described above. Knockout efficiency was confirmed by Western blotting. All experiments were performed 48–72 h after transfection or infection unless otherwise indicated.

**Establishment of surufatinib-Resistant QBC-939 Cells**

To generate a surufatinib-resistant subline (QBC-939/SR), parental cells were first exposed to 0.1 µM surufatinib (Selleck, S0487) for one week. Once the cultures reached 70–80 % confluence with proliferation rates comparable to drug-free controls for two consecutive passages, the surufatinib concentration was increased stepwise (0.1 → 0.2 → 0.5 → 1 → 2 → 5 → 10 µM). Each concentration was maintained for at least two passages before escalation. Culture medium containing the appropriate drug concentration was refreshed every 48 h.

The entire selection process required approximately 3–4 months, culminating in stable growth of QBC-939 cells in 10 µM surufatinib. These cells were subsequently maintained in 10 µM surufatinib for a minimum of four additional weeks, followed by single-cell cloning by limiting dilution. Clonal populations exhibiting robust growth in 10 µM surufatinib were expanded and cryopreserved in liquid nitrogen for long-term storage.

**Xenograft Models**

Female C57BL/6 mice and NOD/SCID mice (5 weeks old, 16–20 g) were purchased from GemPharmatech Co., Ltd. (Nanjing, China). The murine cholangiocarcinoma cell line LD1 (1×10⁶) was orthotopically implanted into the liver of each mouse under anesthesia. Mice were randomly assigned to different treatment groups according to the experimental design. From day 3 post-implantation, treatments were administered once daily via intraperitoneal injection, including: Mouse LR3-IGF-1 (15 mg/kg, i.p., Novoprotein, CR39), linsitinib (30 mg/kg, i.p., Selleck, S1091), surufatinib (20 mg/kg, i.g., Selleck, S0487).

To investigate the role of tumor–stromal interactions, human cholangiocarcinoma QBC939 cells (1×10⁶) were co-injected with primary human CAFs (1×10⁶) or genetically modified CAFs (oeIGF1, sgIGF1, 1×10⁶) into the liver of NOD/SCID mice. In some groups, mice were treated with Human IGF-I/IGF-1 Antibody (5 mg/kg, i.p., R&D Systems, MAB2912) to neutralize CAF-derived IGF1. In addition, LD1 cells were genetically modified to overexpress or knockout IGF1R or PTPN9, followed by treatment with linsitinib (30 mg/kg, i.p., Selleck, S1091), surufatinib (20 mg/kg, i.g., Selleck, S0487) alone or in combination. On day 12 post-implantation for LD1-injected C57BL/6 mice and day 28 post-implantation for QBC939/CAF-injected NOD/SCID mice, animals were sacrificed; livers were excised, photographed, weighed, and processed. Tumor burden was measured post-mortem by weighing the tumors and corresponding livers and calculating the tumor-to-liver weight ratio. All animal experiments were conducted in accordance with institutional guidelines and approved by the Medical Ethics Committee of Shandong University (approval no. KYLL-2021-624).

**Western Blotting**

Total protein was extracted using radioimmunoprecipitation assay (RIPA) lysis buffer (Solarbio, R0010) supplemented with 1% PMSF (Beyotime, ST2573) and 1% phosphatase Inhibitor Cocktail I and II (Apexbio, K1012 and K1013). After denaturation, proteins were separated using 10% SDS-PAGE and transferred to polyvinylidene fluoride (PVDF) membranes (Millipore, IPVH00010). All primary antibodies and secondary antibodies were listed in tables above. The signal was visualized using an enhanced chemiluminescence (Millipore) detection system.

**Co-immunoprecipitation (Co-IP)**

For exogenous Co-IP, HEK293T cells were co-transfected with plasmids encoding HA-tagged IGF1R or empty HA-vector, along with Flag-tagged PTPN9^C515S^ using Lipofectamine 3000 (Invitrogen, L3000015), according to the manufacturer’s instructions. After 24 hours, cells were treated with or without IGF1 (50 ng/mL) for 15 minutes before harvesting. Cells were lysed in ice-cold NP-40 lysis buffer (50 mM Tris-HCl pH 7.4, 150 mM NaCl, 1% NP-40, 1 mM EDTA, protease and phosphatase inhibitors). Lysates were incubated with ANTI-FLAG® M2 Affinity Gel (sigmaaldrich, A2220) or-Anti-HA Magnetic Beads (MCE, HY-K0201) overnight at 4°C. Immunoprecipitates were washed three times with lysis buffer and subjected to SDS-PAGE followed by immunoblotting with anti-HA and anti-Flag antibodies to detect co-precipitated HA-IGF1R and Flag-PTPN9^C515S^, respectively.

For endogenous Co-IP, QBC939 and RBE cells were lysed using the same NP-40 buffer. Cell lysates were incubated overnight at 4°C with anti-PTPN9 antibody (Proteintech, 12109-1-AP) or anti-IGF1R antibody (Proteintech, 20254-1-AP), followed by incubation with Protein A/G magnetic beads (MCE, HY-K0202) for 2 hours. Normal rabbit IgG was used as a negative control. After extensive washing with lysis buffer, bound proteins were eluted by boiling in SDS sample buffer and analyzed by immunoblotting using anti-IGF1R (Proteintech, 20254-1-AP) and anti-PTPN9 (Proteintech, 12109-1-AP) antibodies.

**Immunofluorescence**

Paraffin-embedded human CCA tissue sections were deparaffinized in xylene and rehydrated through a graded series of ethanol. Antigen retrieval was performed by boiling the sections in citrate buffer (pH 6.0) for 20 minutes. After cooling to room temperature, sections were blocked with 5% BSA (Beyotime, ST2254) in PBS containing 0.2% Triton X-100 (Sigma, T8787) for 1 hour at room temperature.

For co-staining of IGF1R and PTPN9, tissue sections were incubated overnight at 4°C with anti-IGF1R antibody (Abcam, ab131476) and anti-PTPN9 antibody (Proteintech, 67931-1-Ig). After washing, sections were incubated with appropriate Alexa Fluor 488 (Abcam, ab150113) - and Alexa Fluor 594 (Abcam, ab150080)-conjugated secondary antibodies for 1 hour at room temperature in the dark. Nuclei were counterstained with DAPI (Abcam, ab104139) for 5 minutes.

QBC939 and CAFs(P1–P2) were seeded on 12-mm glass coverslips placed in 24-well plates at 3×10^4^ cells/well and allowed to attach overnight. Cells were rinsed with PBS and fixed in 4% PFA for 10 min at RT, followed by permeabilization with 0.1% Triton X-100 in PBS for 10 min. Non-specific binding was blocked with 5% BSA in PBS for 1 h at RT. Coverslips were incubated with primary antibodies diluted in blocking buffer overnight at 4 °C: anti-α-SMA(Cell Signaling Technology, 19245), anti-FAP(Invitrogen, PA5-99458), anti-E-cadherin(Proteintech, 20874-1-AP), and anti-pan-Cytokeratin(Invitrogen, MA5-13203). After three PBS washes, species-appropriate secondary antibodies conjugated to Alexa Fluor 594 were applied for 1 h at RT in the dark. Nuclei were counterstained with DAPI (Abcam, ab104139) for 5 minutes. Slides were mounted using antifade mounting medium and imaged with a confocal laser scanning microscope (Carl Zeiss, LSM880). Images were processed using ImageJ software.

**Tissue Microarray and Immunohistochemistry (IHC)**

Typical paraffin-embedded sections of cholangiocarcinoma (CCA) tissues were used for tissue microarray (TMA) construction and immunohistochemistry (IHC) scoring. Hematoxylin and eosin (H&E) staining was performed to confirm the histological features of all samples, which were reviewed by a senior pathologist. For TMA construction, a 1.5 mm diameter core biopsy was extracted from each sample and placed onto a TMA slide.

Prior to IHC, antigen retrieval was performed using either EDTA buffer (Beyotime, C0196) or sodium citrate buffer (pH 6.0). Primary antibody incubation was conducted at 4°C overnight using anti-PTPN9 (Proteintech, 12109-1-AP), anti-IGF1R (Proteintech, 20254-1-AP), and anti-phosphorylated IGF1R (Invitrogen, PA5-37602). Subsequently, biotin-labeled rabbit anti-goat and goat anti-rabbit antibodies (Zsbio, Beijing, China) were applied and incubated at room temperature for 30 minutes. The peroxidase reaction was visualized using 3,3’-diaminobenzidine (DAB) solution (Coolaber).

The stained slides were scanned using a slide scanner. The tumor area was selected by a senior pathologist and IHC results were quantified using Quant Center software. The area of each staining was calculated, and staining intensity was classified into weak, medium, or strong. The IHC score was determined using the formula: IHC score = (percentage of weak intensity × 1) + (percentage of medium intensity × 2) + (percentage of strong intensity × 3). The study cohort was divided into different groups based on the IHC score median value, where samples with an IHC score less than or equal to the median were classified as low expression, while those above the median were classified as high expression.

**H&E staining**

Tumor-bearing liver tissues were harvested, fixed in 4% paraformaldehyde overnight at 4°C, embedded in paraffin, and sectioned at 4 μm thickness. Tissue sections were deparaffinized, rehydrated, and stained with hematoxylin and eosin (H&E) using standard procedures. Images were captured using a light microscope (Carl Zeiss, LSM880).

**ELISA**

The secretion level of IGF1 in the conditioned media (CM) from various cell types was quantified using a human IGF1 ELISA kit (R&D SYSTEMS, DG100B) according to the manufacturer’s instructions. Briefly, cells were cultured in serum-free medium for 24 hours. The collected CM was centrifuged to remove cell debris and applied to ELISA plates precoated with anti-IGF1 antibodies. After incubation with detection antibodies and substrate solution, absorbance at 450 nm was measured using a BIOTEK spectrophotometer (Vermont, USA). For experiments involving CAFs with IGF1 knockout (CAF-sgIGF1) or overexpression (CAF-oeIGF1), cells were seeded at equal densities and cultured under identical conditions. CM was harvested and analyzed for IGF1 secretion using the same ELISA protocol. To account for differences in cell numbers, IGF1 concentrations were normalized to total cell counts. At the time of CM collection, adherent cells were detached using trypsin-EDTA and counted using a hemocytometer. Final IGF1 levels were expressed as pg/mL per 10⁶ cells.

**Flow cytometry**

For flow cytometry, early-passage CAF cells (P1–P2) were detached with Accutase, washed, and resuspended in FACS buffer (PBS, 2% FBS, 2 mM EDTA). After Fc-receptor blocking (10 min, 4 °C), cells were stained for 30 min at 4 °C in the dark with the following fluorochrome-conjugated antibodies: anti-CD45(Invitrogen, 58-0459-42), anti-EpCAM (Invitrogen, 11-5791-82), and anti-PDGFRα (Invitrogen, 17-1401-81). A fixable Live/Dead dye (7-AAD/DAPI) and matched isotype controls were included. Single-color compensation controls were prepared using antibody-capture beads.

Data were acquired on a BD flow cytometer and analyzed with FlowJo v10. The gating strategy was: (i) cells by FSC-A/SSC-A to exclude debris; (ii) singlets by FSC-H vs. FSC-A; (iii) live cells by viability dye negative; (iv) lineage exclusion CD45⁻/EpCAM⁻; and (v) fibroblast gate PDGFRα⁺. Positivity thresholds were set using isotype controls. Purity was defined as the percentage of PDGFRα⁺ cells within the live singlet CD45⁻/EpCAM⁻ population.

**Mass Spectrometry and Interactome Analysis**
HEK293T cells were transfected with plasmids encoding vector control, PTPN9^C515S^, or PTPN9^D470A^, each fused with a FLAG-tag. After 48 hours, cells were lysed in IP lysis buffer (Beyotime, P0013) supplemented with protease and phosphatase inhibitors. FLAG-tagged proteins and their interacting partners were immunoprecipitated using anti-FLAG M2 magnetic beads (Sigma-Aldrich, M8823) at 4°C overnight. After extensive washing, bound proteins were eluted and subjected to SDS-PAGE followed by silver staining. Gel slices containing the protein complexes were excised and digested with trypsin for liquid chromatography-tandem mass spectrometry (LC-MS/MS) analysis.

Peptides were analyzed using a high-resolution Orbitrap mass spectrometer (Thermo Scientific Q Exactive). The resulting spectra were searched against the UniProt human protein database using Mascot or MaxQuant software. Protein identifications were filtered using a 1% false discovery rate. Shared and specific interactors among different groups were visualized using Venn diagrams. Peptide hits for key interactors were quantified and ranked based on spectral counts.

**Site-directed mutagenesis of specific amino acid site**

Site-directed mutagenesis of PTPN9 variants (Y333A, D335A, Y471A, S516A, S519A, S556A, S559A, D470A and C515S) and IGF1R mutants (Y1165/66E and Y1165/66F) were performed using primers designed with NEBasechanger (New England Bioloabs), the KOD -Plus- Mutagenesis Kit (Toyobo, SMK-101) and NEB Stable competent high-efficiency E. coli (New England Biolabs, C3040H).

**In vitro PTPN9 phosphatase assay**

Enzymatic activity measurement was carried out as previously reported (Xu et al, 2021). The standard solution (DMG buffer) for our enzymatic reactions is as follows: 50 mM 3, 3-dimethyl glutarate pH 7.0, 1 mM EDTA, and 1 mM DTT. The ionic strength was maintained at 0.15 M (adjusted by NaCl). For the pNPP activity measurement, 100 µl reaction mixtures were set up in a total volume in a 96-well polystyrene plate (Thermo Fisher Scientific, Waltham, MA, US). Reactions were started by the addition of an appropriate amount of GST-PTPN9 or corresponding mutants, such as Y333A, D335A, Y471A, S516A, S519A, S556A, S559A. The dephosphorylation of pNPP was terminated by adding 120 µl 1 M NaOH, and the enzymatic activity was monitored by measuring the absorbance at 405 nm. The activities toward phospho-peptide segment derived from IGF1R-pY1165/pY1166 were measured as following: Diluted IGF1R-pY1165/pY1166 phospho-peptide substrate (1600, 1200, 1000, 800, 600, 400, 200, 100, 50, 25, 12.5, 6.25, 3.125, 1.5625uM) was added into each column and pre-incubated at 37°C for 5 min. Reactions were started by the addition of an appropriate number of enzymes (GST-PTPN9 or corresponding mutants). The dephosphorylation of IGF1R-pY1165/pY1166 was terminated by adding 120 µl Malachite Green Reagent (Beyotime, S0196), and the enzymatic activities were monitored by measuring the absorbance at 620 nm. Enzymatic kinetic curves, K_m_ and K_cat_ values, and catalytic efficiencies (K_cat_/K_m_) for different phospho-peptide substrates were calculated by non-linear regression fitting to the Michaelis–Menten model and visualized using Python (v3.13, matplotlib).

**Collection of conditioned culture medium**

Briefly, CAFs were first cultured in DMEM supplemented with 10% FBS until reaching 80–90% confluence. The medium was then replaced with low-serum medium (1% FBS) for 24 hours to allow cytokine secretion. The CM was collected, centrifuged at 2000 rpm for 5 minutes to remove cell debris, the resulting supernatant was filtered through a low-protein-binding 0.2 μm filter and concentrated using Amicon Ultra 15 mL filters. The CM was finally collected and stored at -80°C for further use.

**Colony Formation Assay**

Cells were seeded into 6-well plates at a density of 1000 cells per well in 2 mL of complete culture medium. According to the experimental conditions, IGF1 (50ng/ml, Novoprotein, DC031), surufatinib (10μM, Selleck, S0487) or linsitinib (10μM, Selleck, S1091) were added to the culture medium. The plates were gently shaken to ensure even distribution of cells and incubated at 37°C with 5% CO₂. After 24 hours, allowing for cell attachment, the medium was refreshed, and cells were maintained for 12 days, with the medium replaced every 3 days until visible colonies formed.

For treatment with culture medium, tumor cells were seeded under the same conditions as in the standard assay and incubated for 24 hours to allow attachment. Subsequently, the cells were stimulated with concentrated and conditioned medium from CAFs, while the control group was switched to the same batch of concentrated low-serum basal medium that had been used for CAF conditioning but without prior CAF exposure, and Human neutralizing IGF-I/IGF-1 Antibody (1μg/ml, R&D systems, MAB2912) or surufatinib (10μM, Selleck, S0487) was added as required. Control wells received RPMI-1640 containing 1% FBS (medium-only control). Cells were subsequently maintained under the same conditions as the standard assay until colonies formed.

Once colonies formed in both assays, the medium was removed, and cells were washed twice with PBS. Colonies were fixed with 4% paraformaldehyde (Beyotime, P0099) for 15 minutes at room temperature, followed by two additional PBS washes. Cells were then stained with 0.1% crystal violet solution (Beyotime, C0121) for 15 minutes and gently rinsed with running water to remove excess stain. After air-drying, colony formation was observed and photographed.

**CCK8 Assay**

Cells were seeded into 96-well plates at a density of 2×10³ cells per well and incubated for 6 hours to allow attachment. Baseline absorbance at 450 nm (OD450) was measured using a BIOTEK spectrophotometer (Vermont, USA) after the addition of 100 μL of CCK8 reagent (Targetmol, C0005) and a 1-hour incubation at room temperature.

For the standard assay, the culture medium was replaced with fresh medium containing

IGF1 (50ng/ml, Novoprotein, DC031), surufatinib (10μM, Selleck, S0487) or linsitinib (10μM, Selleck, S1091) according to the experimental design. The drug-containing medium was refreshed every other day, and cell proliferation was measured daily for 5 consecutive days using the CCK8 assay, following the same incubation and absorbance measurement protocol as baseline.

For treatment with culture medium, tumor cells were seeded and baseline OD450 was measured following the same protocol as the standard assay. Subsequently, the cells were stimulated with concentrated and conditioned medium from CAFs, while the control group was switched to the same batch of concentrated low-serum basal medium that had been used for CAF conditioning but without prior CAF exposure, with Human neutralizing IGF-I/IGF-1 Antibody (1μg/ml, R&D systems, MAB2912) or surufatinib (10μM, Selleck, S0487) was added as required. Control wells received RPMI-1640 containing 1% FBS (medium-only control). Cell proliferation was assessed daily for 5 days using the CCK8 assay.

**Transwell Assay**

Transwell assays were conducted to assess cell migration and invasion using 24-well plates with chambers containing 8.0-μm pore membranes (Corning, 3464). Depending on the experimental design, the membranes were either pre-coated with 50 µL Matrigel (1mg/mL, Corning, 356234) at 37 °C for 2 h, or left uncoated. For both standard and co-culture assays, RBE or QBC-939 cells (5×10⁴) were suspended in 200 μL serum-free RPMI 1640 medium and seeded into the upper chamber. The lower chamber was filled with 600 μL medium, which varied by condition: in the standard assay, it contained RPMI 1640 supplemented with 20% FBS as a chemoattractant, with IGF1 (50ng/ml, Novoprotein, DC031) , surufatinib (10μM, Selleck, S0487) or linsitinib (10μM, Selleck, S1091) added according to the experimental setup; in the co-culture assay, CAFs were cultured in complete medium until ~80–90 % confluence. The medium was then replaced with 600 µL RPMI-1640 containing 1 % FBS; for conditions using THP-1 or SU-DHL-4, the lower chamber was loaded with THP-1 or SU-DHL-4 at 0.5–1.0 × 10^6^ cells/mL (600 μL per well) in the same RPMI-1640/1% FBS. Human IGF-1 neutralizing antibody (1 µg/mL, R&D, MAB2912) or surufatinib (10μM, Selleck, S0487) was added as required. Control wells contained 600 μL RPMI-1640 with 1% FBS without cells (medium-only control). Tumor cells (5 × 10⁴) in 200 µL serum-free medium were seeded into the upper chamber immediately thereafter. After incubation at 37°C for 24 hours, non-migrated cells on the upper side of the membrane were carefully removed with a cotton swab. Migrated or invaded cells on the lower side were fixed with methanol, stained with 0.5% crystal violet for 30 minutes, washed three times with PBS, and counted in six randomly selected fields at 200× magnification.

**Quantitative real-time PCR**

Total mRNA was isolated from frozen CCA tissues using TRIzol reagent (Invitrogen) according to the manufacturer's instructions. RNA quality was identified by NanoDrop One (Thermo Fisher). Purified RNA was reverse-transcribed into cDNA using the high-capacity cDNA Reverse Transcription Kit (TOYOBO, Japan), and RT-qPCR was performed using SYBR Green master mix (Roche, Switzerland). The relative expression levels of the target genes were calculated using the 2-ΔΔCt method, with *ACTB* serving as the internal control. The primer sequences were designed using PrimerBank (https://pga.mgh.harvard.edu/primerbank/). The forward and reverse primer sequences for *IGF1* were GCTCTTCAGTTCGTGTGTGGA and GCCTCCTTAGATCACAGCTCC, respectively, while the forward and reverse primer sequences for *ACTB* were CATGTACGTTGCTATCCAGGC and CTCCTTAATGTCACGCACGAT, respectively.

**In Silico Analysis**

Transcriptomic data of cholangiocarcinoma (CHOL) was obtained from The Cancer Genome Atlas (TCGA-CHOL) database. The volcano plot and expression dot plots were generated using Python (v3.13) scripts executed in Visual Studio Code (VS Code, Microsoft), utilizing the pandas, numpy, matplotlib, and seaborn libraries. Single-cell RNA sequencing (scRNA-seq) data from three pCCA samples, four dCCA samples, and four tumor-adjacent tissues were obtained from the GEO database (GSE213452). These were integrated with published scRNA-seq data from intrahepatic cholangiocarcinoma (iCCA) samples (GSE138709) for downstream analysis. The R package Seurat (v4.3.0) was used for data integration, dimensionality reduction (UMAP), clustering, and visualization. A cell-type–specific heatmap of the IGF/IGF1R family genes was also generated to characterize fibroblast-specific IGF1 expression. Kaplan–Meier survival analysis and multivariate Cox regression were conducted using Python (v3.13, lifelines, matplotlib) to evaluate prognostic significance.

All in silico and bioinformatics analyses were carried out using default parameters unless otherwise specified. The threshold for significantly differentially expressed genes was set at log₂(fold change) ≥ 4 and P-value < 0.05.

**Bioinformatic search of PTP–protein interactions**

Amino acid sequences of the activation loop regions in human TrkA (UniProt ID: P04629-2), FGFR2 (P21802-1), and IGF1R (P08069) were retrieved from the UniProt database. The sequences containing the conserved dual tyrosine phosphorylation motifs were manually aligned based on position and sequence similarity. Amino acids were categorized by their physicochemical properties into polar acidic, polar uncharged, hydrophobic, and polar basic groups. Classification and color annotation were applied accordingly for comparative visualization.

**Statistical Analysis**

Statistical analyses were performed using R, Python, and GraphPad Prism 9.0 (GraphPad Software, CA). Kaplan–Meier survival curves were generated using the Kaplan–Meier method, with significance assessed by the log-rank test. Univariate and multivariate Cox proportional hazards regression analyses were conducted using Python (version 3.13) with the lifelines package. Overall survival time (OS) and survival status were used as outcome variables. For multivariate analysis, all selected covariates were included in a single Cox regression model using the CoxPHFitter function. IGF1R and PTPN9 expression levels were categorized into high and low expression groups based on the median IHC score. Tumor size and age were treated as continuous variables. T stage (T1 and T2 as 0, T3 and above as 1) and N stage (N0 as 0, N1 and above as 1) were coded as binary categorical variables. Tumor differentiation was classified as 1 for poorly differentiated and 0 for well and moderately differentiated cases. Microvascular invasion (MVI) and nerve invasion were binary variables (1 for present, 0 for absent). Gender was also coded as a binary variable (0 for female, 1 for male). Categorical variables were converted into appropriate dummy variables where necessary. Hazard ratios (HRs) and 95% confidence intervals (CIs) were calculated by exponentiating the regression coefficients. Forest plots were generated using the matplotlib package to visually present the prognostic effect of each variable. The unpaired t-test and paired t-test were used to compare differences between two groups, as appropriate. Differences among multiple groups were analyzed using two-way ANOVA. The chi-square (χ²) test was applied to assess categorical variables. Correlation between IHC scores was assessed using Pearson’s correlation coefficient, and scatter plots with regression lines were generated using Python (matplotlib, seaborn). A p-value < 0.05 was considered statistically significant.

**Clinical Imaging Data Collection**
Representative pre- and post-treatment CT images of CCA patients were obtained retrospectively from the radiology database of Qilu Hospital of Shandong University. Patients were classified as surufatinib-response or -non-response based on radiological response following treatment. Tumor boundaries were manually outlined for illustration purposes. This study was conducted in accordance with the ethical principles of the 2013 Declaration of Helsinki and the 2018 Declaration of Istanbul. All patients provided written informed consent, and the study was approved by the Institutional Review Board of Qilu Hospital of Shandong University (IRB no. 2021176).

**scRNA-seq Data Processing**

We processed the raw data using Seurat (v5.1.0) and applied stringent quality control criteria, retaining cells with 200–6000 detected genes, <20% mitochondrial gene content, <50% ribosomal gene content, and <2% hemoglobin gene content to remove low-quality cells and potential doublets. Dimensionality reduction was performed using principal component analysis (PCA; top 50 PCs), followed by batch-effect correction with Harmony and two-dimensional visualization with UMAP. Clustering was conducted using the Louvain algorithm, and after evaluating multiple parameters, we selected a resolution of 1.5, which yielded 51 transcriptionally distinct clusters for downstream analyses. For integration, we applied Harmony on the PCA embeddings using dataset and sample ID as batch covariates; the Harmony-corrected embeddings (PCs 1–50) were then used for downstream clustering and UMAP. To avoid integration-induced bias, all differential expression analyses for marker identification were performed on the non-integrated RNA assay. No further subclustering was applied. Cell type annotation was based on canonical marker genes as follows: Monocytes/macrophages: FCN1, LILRA5, FPR1, TREM2, CD300E. T/NK cells: CD3G, TRAC, TRBC1, CD2, IFNG, CD3D/E, NKG7, KLRD1. B cells: IGLC2/3, IGHG1/2, IGLL5, MS4A1, CD79A/B. Mast cells: TPSAB1, TPSB2, CPA3, MS4A2, TPSD1. Endothelial cells: CLDN5, EMCN, MMRN1, SOX17, MYCT1, PECAM1, VWF, KDR. Fibroblasts: ASPN, FRZB, COL1A1, COL1A2, COL3A1, DCN, LUM, PDGFRA. Epithelial cells: PSCA, FAM83A, FXYD3, MIR205HG, KRT16, EPCAM, KRT8, KRT18.

**Details of antibodies and reagents are as follows:**

| **Antibodies** | **SOURCE** |  | **IDENTIFIER** |
| --- | --- | --- | --- |
| Phospho-IGF-I Receptor β (Tyr1135/1136) | CST |  | Cat. No. 3024 |
| IGF1R Polyclonal antibody | Proteintech |  | Cat. No. 20254-1-AP |
| PTPN9 | Proteintech |  | Cat. No. 12109-1-AP |
| IGF1 | Proteintech |  | Cat. No. 28530-1-AP |
| Phospho-IGF1R beta (Tyr1165, Tyr1166) Polyclonal Antibody | Invitrogen |  | Cat. No. PA5-37602 |
| Phospho-VEGF Receptor 2 (Tyr1175) | CST |  | Cat. No. 2478 |
| VEGF Receptor 2 | CST |  | Cat. No. 9698 |
| Phospho-Akt (Thr308) | CST |  | Cat. No. 13038 |
| Akt Antibody | CST |  | Cat. No. 9272 |
| ERK2 | Selleck |  | Cat. No. F1637 |
| p44/42 MAPK (Erk1/2) | CST |  | Cat. No. 4695 |
| α-Smooth Muscle Actin | CST |  | Cat. No. 19245 |
| Anti-IGF1 Receptor | abcam |  | Cat. No. ab131476 |
| PTPN9 | Proteintech |  | Cat. No. 67931-1-Ig |
| IGF1R Monoclonal antibody | Proteintech |  | Cat No. 66283-1-Ig |
| GAPDH | Proteintech |  | Cat. No. 60004-1-Ig |
| Beta Actin Monoclonal antibody | Proteintech |  | Cat. No. 66009-1-Ig |

***Continued：***

| **Antibodies** | | **SOURCE** |  | **IDENTIFIER** | |
| --- | --- | --- | --- | --- | --- |
| HSP90 Polyclonal antibody | | Proteintech |  | Cat. No. 13171-1-AP | |
| FAP Polyclonal Antibody | | Invitrogen |  | Cat. No. PA5-99458 | |
| E-cadherin Polyclonal antibody | | Proteintech |  | Cat. No. 20874-1-AP | |
| anti-pan-Cytokeratin | | Invitrogen |  | Cat. No. MA5-13203 | |
| Horizon™ BUV395 Mouse Anti-Human CD45 | | BD biosciences |  | Cat. No. 563792 | |
| PE/Cyanine7 anti-human CD140a (PDGFRα) Antibody | | BioLegend |  | Cat. No. 323508 | |
| EPCAM/CD326 Polyclonal antibody | | Proteintech |  | Cat. No. 21050-1-AP | |
| Goat Anti-Mouse IgG (H+L) | | CST |  | Cat. No. 7076 | |
| Goat Anti-Rabbit IgG (H+L) | | CST |  | Cat. No. 7074 | |
| **Peptides** | **Peptide sequence** | | |  | **Source** |
| p-IGF1R^Y1165/Y1166^ | TYETD**p-Yp-Y**RKGGK | | |  | Synpeptide |
| p-IGF1R^Y1165^ | IYETD**p-Y**YRKGGK | | |  | Synpeptide |
| p-IGF1R^Y1166^ | TYETDY**p-Y**RKGGK | | |  | Synpeptide |
| p-NSF^Y83^ | IEVSL**p-Y**TFDKA | | |  | Synpeptide |
| **Recombinant proteins** | | **SOURCE** |  | **IDENTIFIER** | |
| Human IGF-I | | Novoprotein |  | Cat. No. DC031 | |
| Mouse LR3-IGF-1 | | Novoprotein |  | Cat. No. CR39 | |
| Human IGF-I/IGF-1 Antibody | | R&D systems |  | Cat. No. MAB2912 | |

***Continued：***

| **Recombinant proteins** | **SOURCE** |  | **IDENTIFIER** |
| --- | --- | --- | --- |
| Linsitinib | Selleck |  | Cat. No. S1091 |
| Surufatinib | Selleck |  | Cat. No. S0487 |
| Matrigel | Corning |  | Cat. No. 356234 |
| Anti-Flag Magnetic Beads | MCE |  | Cat. No. HY-K0207 |
| Lipofectamine 3000 | Invitrogen |  | Cat. No. L3000015 |
| Protein A/G Magnetic Beads | MCE |  | Cat. No. HY-K0202 |
|  |  |  |  |

| **Oligonucleotides** | **Target sequences** |
| --- | --- |
| sgPTPN9-1 (human) | AGCCACATTCCAAGACAGCG |
| sgPTPN9-2 (human) | CCACAAGTCAGTCCAACATG |
| sgIGF1R-1(human) | GGAGAACGACCATATCCGTG |
| sgIGF1R-2 (human) | CCTGAGGAACATTACTCGGG |
| sgIGF1-1(human) | CGCCAGGTAGAAGAGATGCG |
| sgIGF1-2(human) | GCTTCCGGAGCTGTGATCTA |
| sgIGF1R(mouse) | ACTCTTCTACAACTACGCAC |
| sgPTPN9-1 (mouse) | TTCTTGAAGAGATCAACAAG |
| sgPTPN9-2 (mouse) | GCGACGTTACCAAGATCTGA |
|  |  |

| **Software** |  |  |
| --- | --- | --- |
| Prism | GraphPad | https://www.graphpad.com/ |
| BioRender | BioRender | https://biorender.com/ |
| R (v4.1.0) | CRAN | https://www.r-project.org/ |
| Python | Python | https://www.python.org/ |
| Caseviewer | Caseviewer | https://www.3dhistech.com/ |


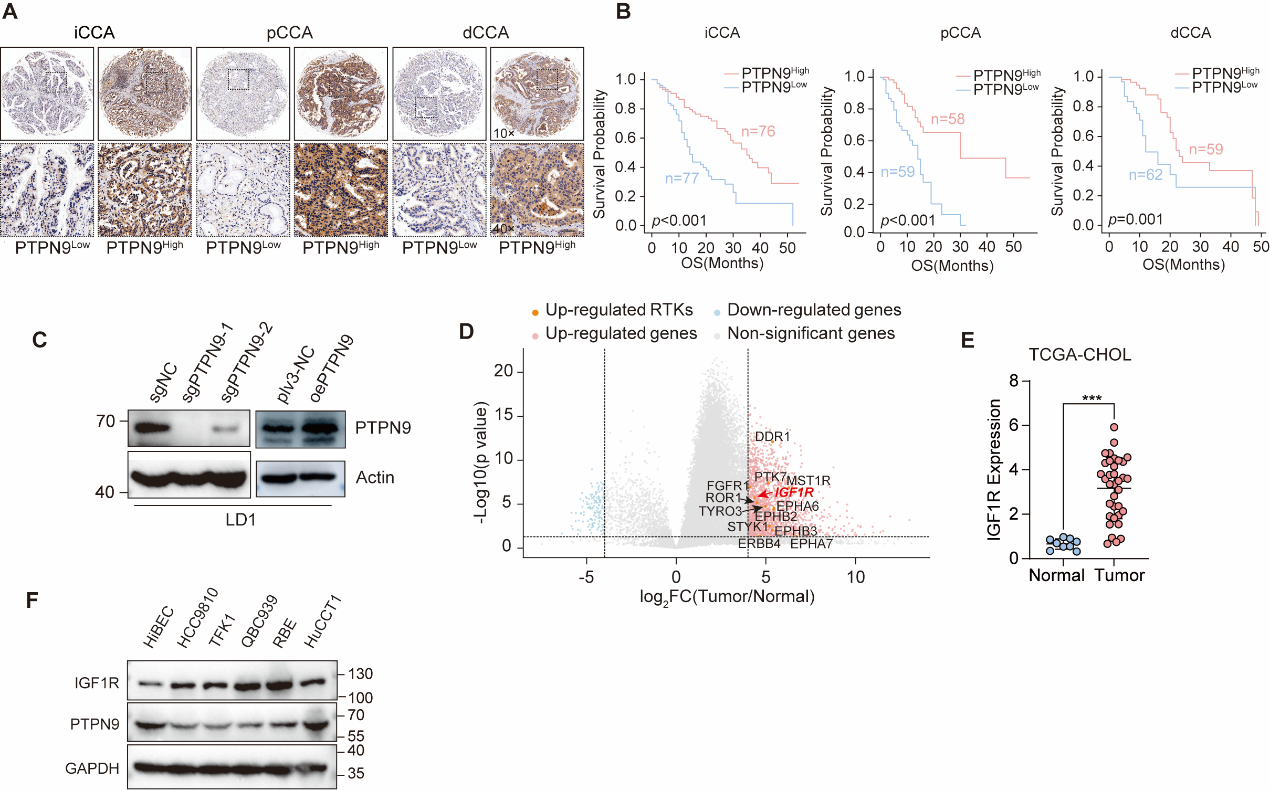


**Supplemental Figure 1 High PTPN9 expression correlates with favorable prognosis of CCA.**

(A) Representative IHC images showing high- and low- PTPN9 expressions in iCCA, pCCA and dCCA tumor tissues. Scale bar: 25 µm. (B) Kaplan–Meier overall-survival curves for iCCA, pCCA and dCCA cohorts. (C) Left: Western blot validation of PTPN9 knockout using sgPTPN9-1 and sgPTPN9-2 in LD1 cells. Right: Western blot confirming oePTPN9 in LD1 cells transduced with lentivirus. (D) Volcano plot of differentially expressed genes in the TCGA‑CHOL dataset. (E) Analysis of IGF1R expression in the TCGA-CHOL dataset. Gene expression values were normalized as TPM and log₂-transformed as log_2_(TPM + 1). (F) Western blot analysis of PTPN9 and IGF1R in CCA cell lines and normal biliary epithelial cells. GAPDH served as the loading control.

Data are from at least three independent experiments and are presented as mean ± SD or representative images. Statistical analyses were performed using log‑rank test (B) and unpaired t-test (C). Significance indicators: ***P < 0.001.


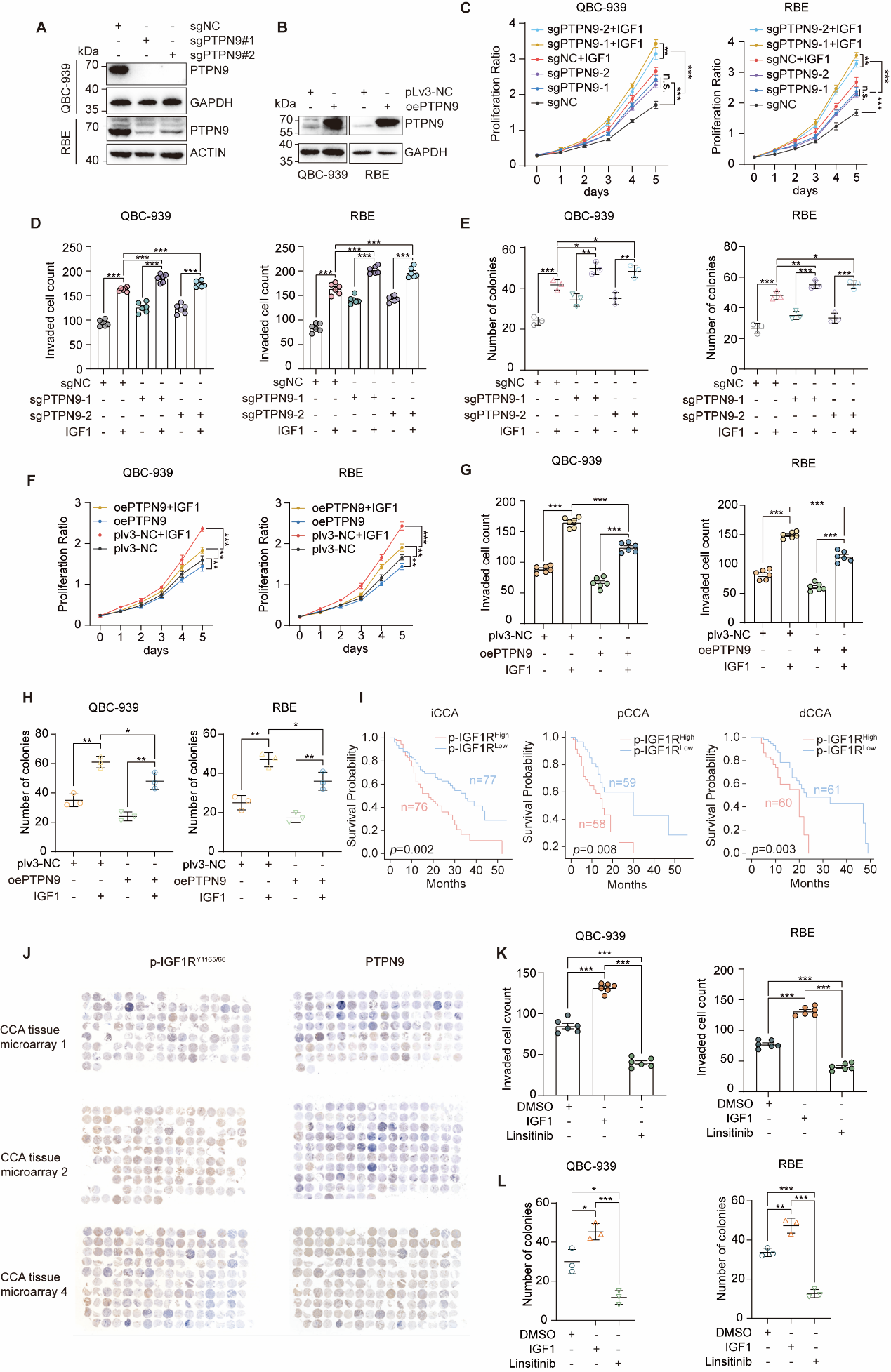


**Supplemental Figure 2. PTPN9 suppresses IGF1-driven proliferation, migration, invasion, and colony formation in CCA cells.**

(A) Western blot validation of PTPN9 knockout using sgPTPN9 in QBC-939 and RBE cells. GAPDH or Actin was used as loading control. (B) Western blot confirming oePTPN9 in QBC-939 and RBE cells transduced with lentivirus. (C) Proliferation assays showing that PTPN9 knockout promotes IGF1-induced cell proliferation in QBC-939 and RBE cells. (D) Transwell invasion assays indicate enhanced motility in sgPTPN9 cells upon IGF1 stimulation in QBC-939 and RBE lines. (E) Colony formation assays showing increased colony numbers upon PTPN9 knockout under IGF1 stimulation in QBC-939 and RBE cells. (F) Proliferation assays showing that PTPN9 over-expression inhibits IGF1-induced cell proliferation in QBC-939 and RBE cells. (G) Over-expression of PTPN9 attenuates IGF1-induced invasion in QBC-939 and RBE cells. (H) Colony formation assays showing decreased colony formation with PTPN9 over-expression under IGF1 stimulation in QBC-939 and RBE cells. (I) Kaplan–Meier overall‑survival curves for iCCA, pCCA, and dCCA cohorts indicate shorter survival in patients with high p-IGF1R expression. (J) Representative TMA scans showing immunohistochemical staining of p-IGF1R and PTPN9 across multiple CCA cohorts. (K) IGF1 promotes invasion in QBC-939 and RBE cells, while Linsitinib reverses these effects. (L) IGF1 promotes colony formation in QBC-939 and RBE cells, while Linsitinib reverses these effects.

Data are from at least three independent experiments and are presented as mean ± SD or representative images. Statistical analyses were performed using two‑way ANOVA (C, F), unpaired t-test (D-G, H, K, L) and log‑rank test (I). Significance indicators: *P < 0.05, **P < 0.01, ***P < 0.001.


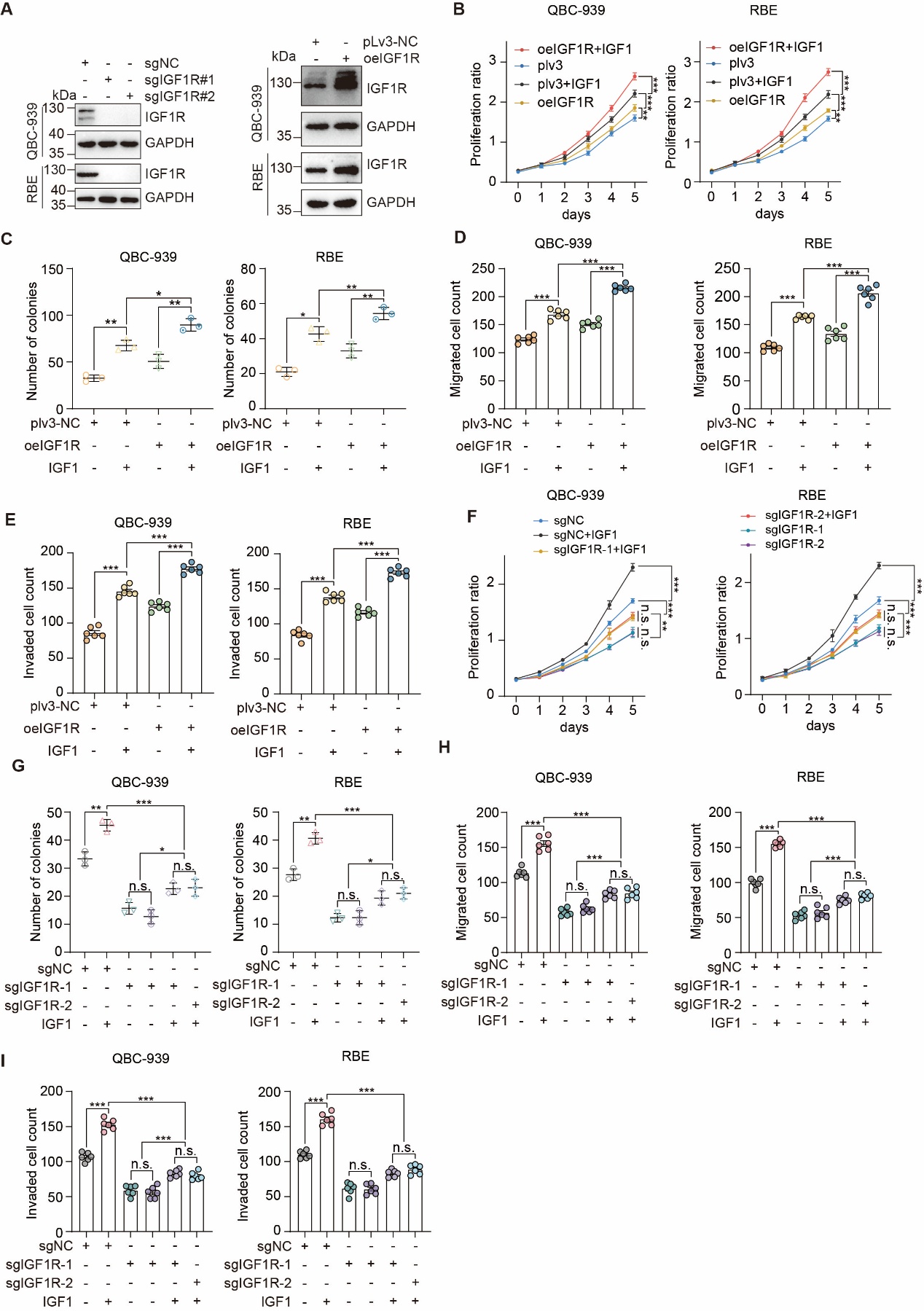


**Supplemental Figure 3. PTPN9 suppresses IGF1-driven proliferation, migration, invasion, and colony formation in CCA cells.**

(A) Western blot validating sgIGF1R (left) and oeIGF1R (right) in QBC-939 and RBE cells; GAPDH served as loading control. (B–E) Over-expression of IGF1R enhances IGF1-induced proliferation (B), colony formation (C), migration (D), and invasion (E) in QBC-939 and RBE cells. (F-I) IGF1R knockout (sgIGF1R-1/2) in QBC-939 and RBE cells suppresses IGF1-driven proliferation (F), colony formation (G), migration (H), and invasion (I) compared to vector controls.

Data are from at least three independent experiments and are presented as mean ± SD or representative images. Statistical analyses were performed using two‑way ANOVA (B, G) and unpaired t-test (C-E, G-I). Significance indicators: *P < 0.05, **P < 0.01, ***P < 0.001.


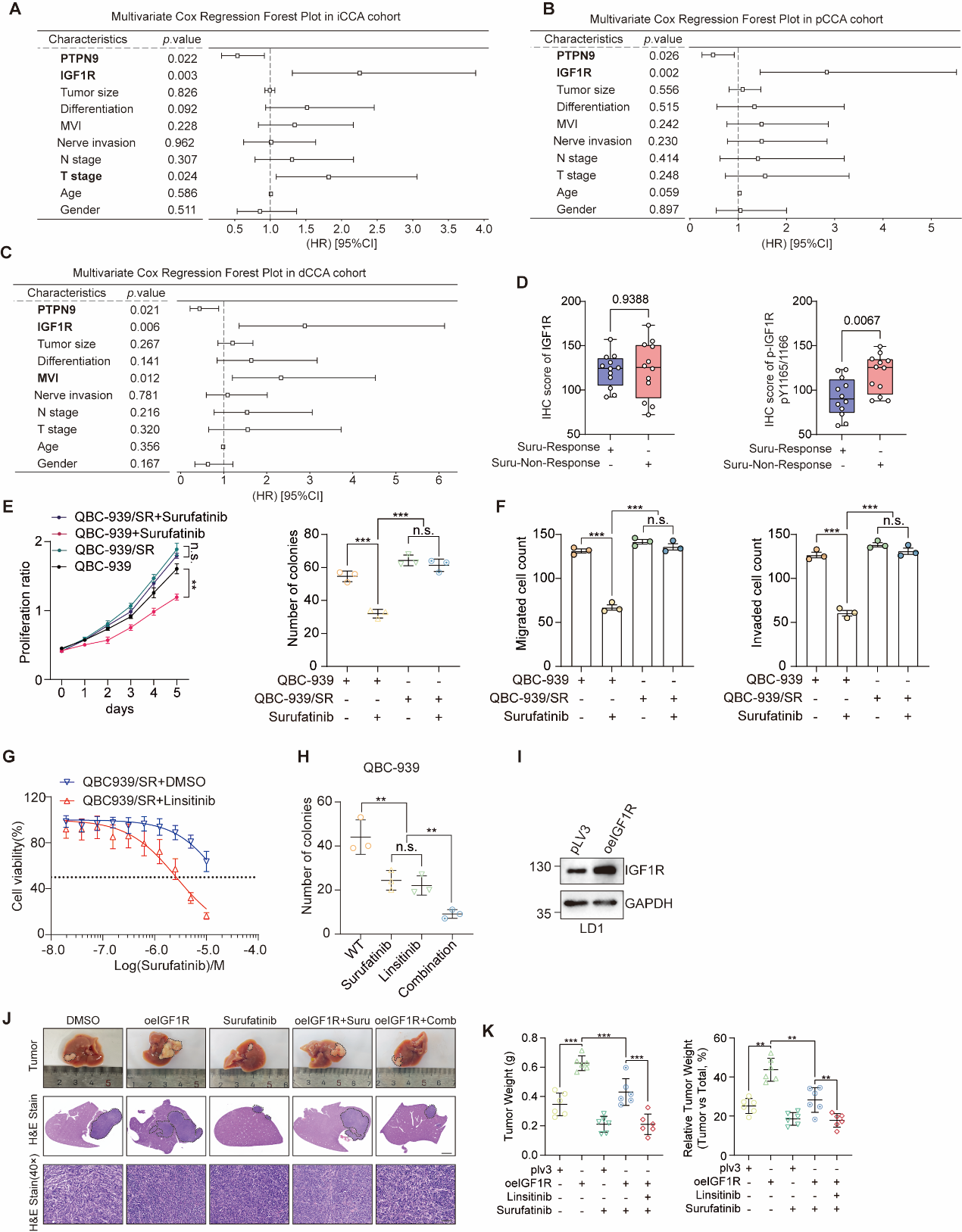


**Supplemental Figure 4. IGF1R bypass activation promotes TKI resistance in CCA.**

(A-C) Multivariate Cox regression forest plot in the iCCA(A), pCCA(B) and dCCA(C) cohort. (D) Quantification of IGF1R and p-IGF1R IHC scores in surufatinib response vs. non-response tumor tissues. (E) Proliferation(left) and Colony formation(right) assays comparing QBC-939 and QBC-939/SR cells with or without surufatinib treatment. (F) Transwell migration (left) and invasion (right) assays comparing QBC-939 and QBC-939/SR cells with or without surufatinib treatment. (G) Dose–response curves showing that Linsitinib restores surufatinib sensitivity in QBC-939/SR cells. (H) Colony formation assay in QBC-939 cells treated with surufatinib, Linsitinib, or combination. (I) Western blot analysis confirming overexpression of IGF1R in murine cholangiocarcinoma LD1 cells. (J) Representative tumor images and H&E‑stained liver sections from orthotopic models (n = 6 per group) receiving DMSO, oeIGF1R, surufatinib, or combination with linsitinib. Tumor regions are outlined with dotted lines. Scale bars: 2 mm (middle panels), 25 µm (lower panels). (K) Tumor weight (left) and relative tumor weight ratio (right) in the orthotopic model.

Data are from at least three independent experiments and are presented as mean ± SD or representative images. Statistical analyses were performed using multivariate Cox regression (A-C), two‑way ANOVA (E) and unpaired t-test (D-F, H, K). Significance indicators: n.s., not significant, *P < 0.05, **P < 0.01, ***P < 0.001.


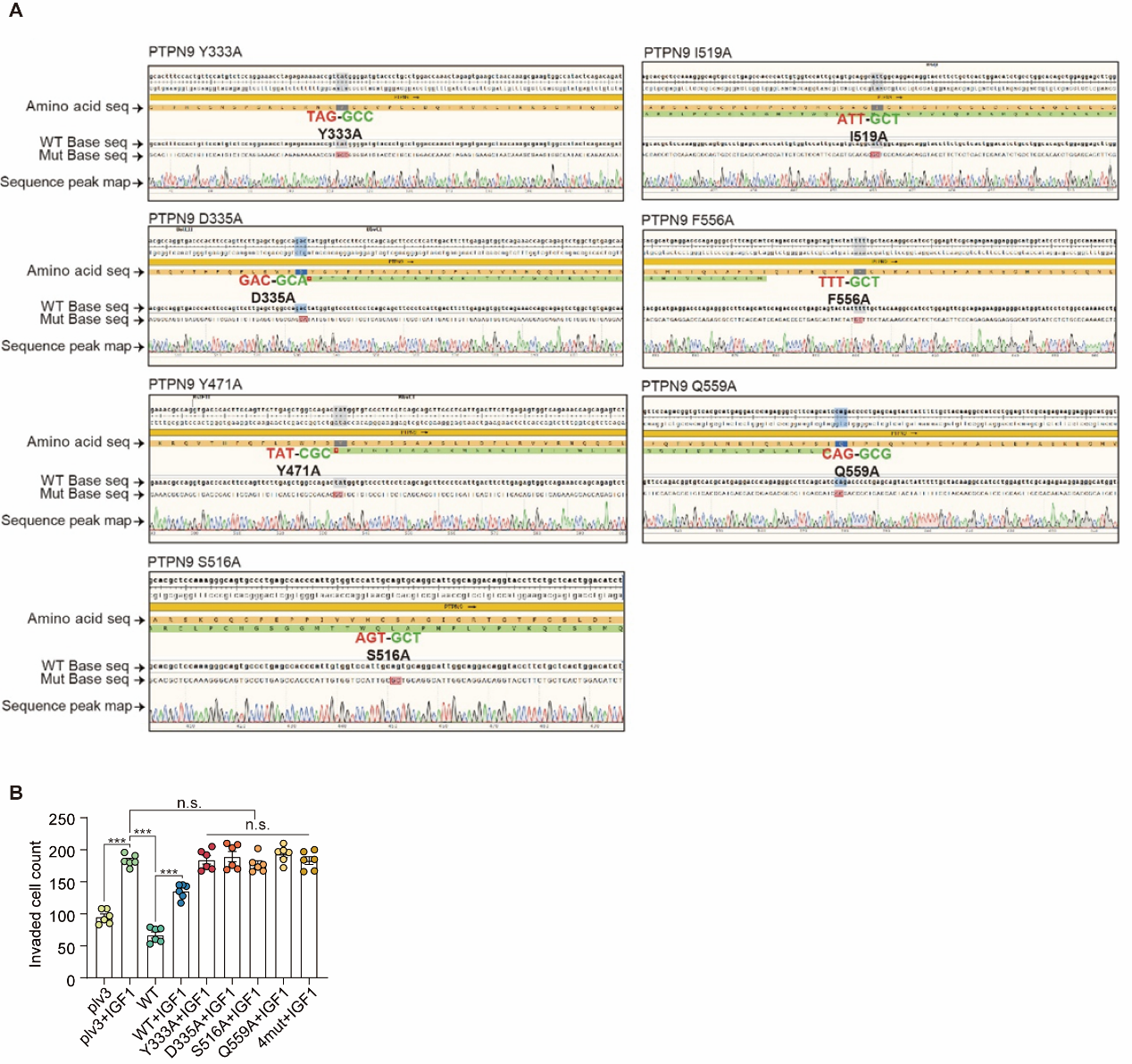


**Supplemental Figure 5.** **PTPN9 Exhibits Substrate Preference and Site-Specific Phosphatase Activity toward IGF1R^Y1165/1166^.**

(A) Verification of site-directed mutagenesis for PTPN9 mutants (Y333A, D335A, Y471A, S516A, I519A, F556A, Q559A,) by Sanger sequencing. (B) Transwell invasion assay of cells overexpressing WT or mutant PTPN9 (Y333A, D335A, S516A, Q559A, or 4mut) in the presence of IGF1.

Data are from at least three independent experiments and are presented as mean ± SD. Statistical analyses were performed using unpaired t-test (B). Significance indicators: n.s., not significant, *P < 0.05, **P < 0.01, ***P < 0.001.


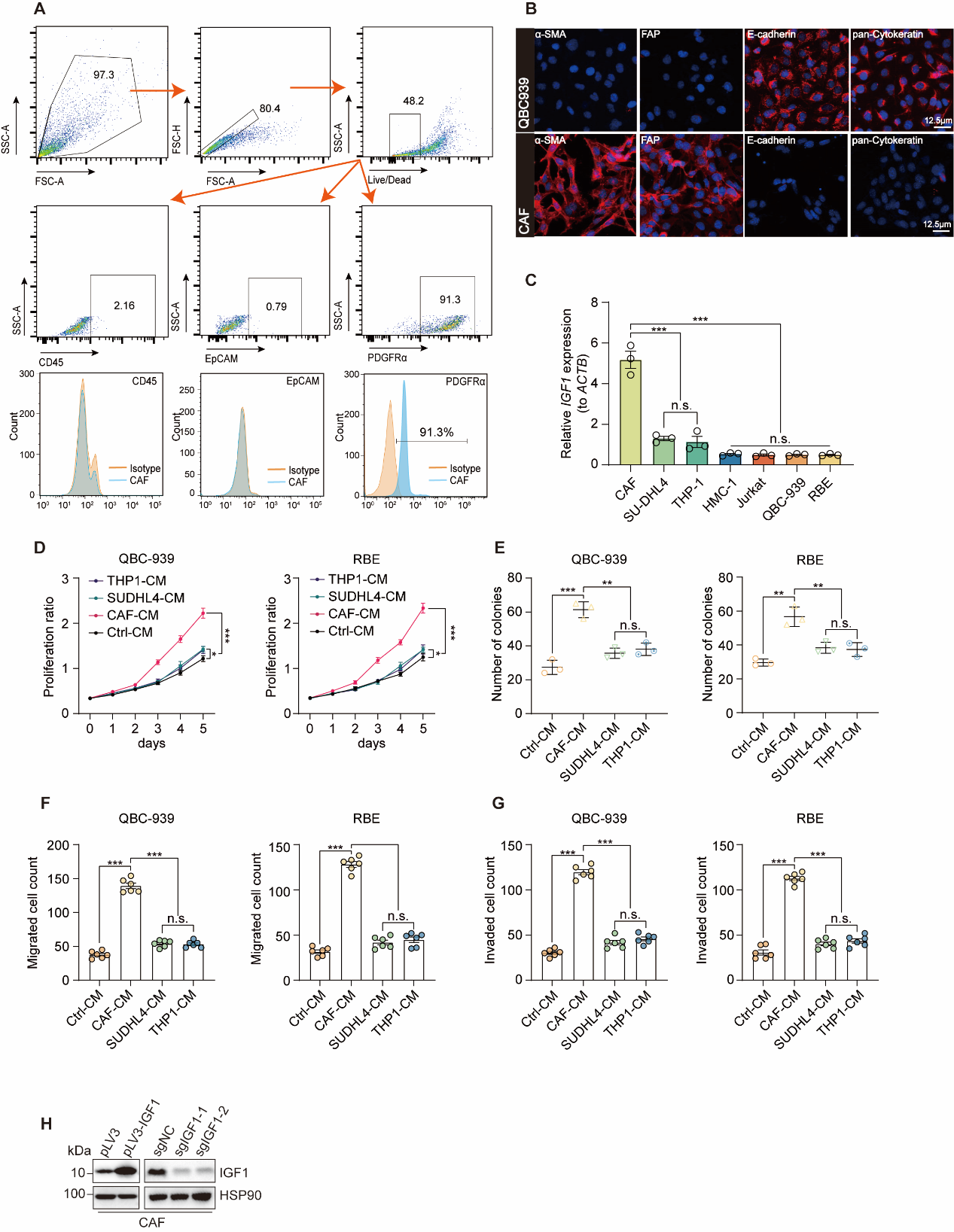


**Supplemental Figure 6. CAF-derived IGF1 activates IGF1R signaling and promotes CCA progression.**

(A) Flow cytometry gating strategy for CAF isolation from cholangiocarcinoma tissue. (B) Immunofluorescence validation of isolated CAFs and QBC-939 tumor cells. (C) IGF1 mRNA levels in multiple cell types. (D–G) Effects of CM from CAF, SU-DHL-4, and THP-1 on CCA cell proliferation (D), colony formation (E), migration (F), and invasion (G). (H) Western blot validating oeIGF1 and sgIGF1 in CAFs; HSP90 served as loading control.

Data are from at least three independent experiments and are presented as mean ± SD or representative images. Statistical analyses were performed using unpaired t‑test (C-E, G) and two‑way ANOVA (D). Significance indicators: n.s., not significant, *P < 0.05, **P < 0.01, ***P < 0.001.


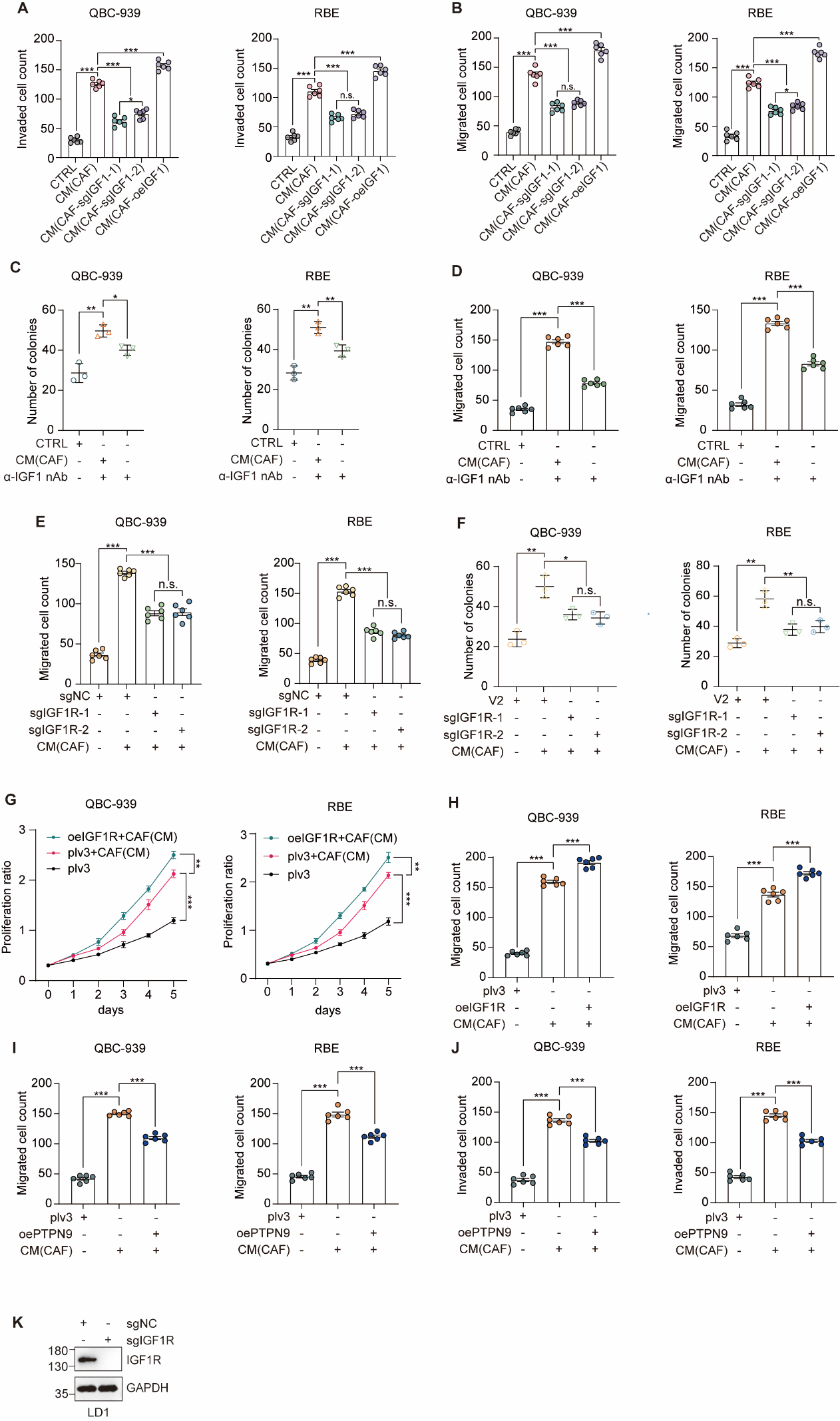


**Supplemental Figure 7. CAF-derived IGF1 activates IGF1R signaling and promotes CCA progression.**

(A, B) Transwell invasion (A) and migration (B) assays in QBC-939 and RBE cells cultured with DMEM or CM from CAFs, sgIGF1 CAFs, or oeIGF1 CAFs. (C, D) Colony formation(C) and migration(D) assays in QBC-939 and RBE cells show enhanced growth with CAF-CM and attenuation by α-IGF1 nAb supplementation. (E, F) Migration (E) and colony formation (F) assays of QBC-939 and RBE cells with sgIGF1R or sgNC. (G, H) Proliferation (G) and migration (H) assay of QBC-939 and RBE cells with oeIGF1R or control vector (plv3). (I, J) Transwell migration (I) and invasion (J) assays of QBC-939 and RBE cells overexpressing PTPN9 or control vector (plv3), cultured with or without CAF-CM. (K) Western blot analysis confirming knockout of IGF1R in murine cholangiocarcinoma LD1 cells.

Data are from at least three independent experiments and are presented as mean ± SD or representative images. Statistical analyses were performed using unpaired t‑test (A-F, H-J) and two‑way ANOVA (G). Significance indicators: *P < 0.05, **P < 0.01, ***P < 0.001.


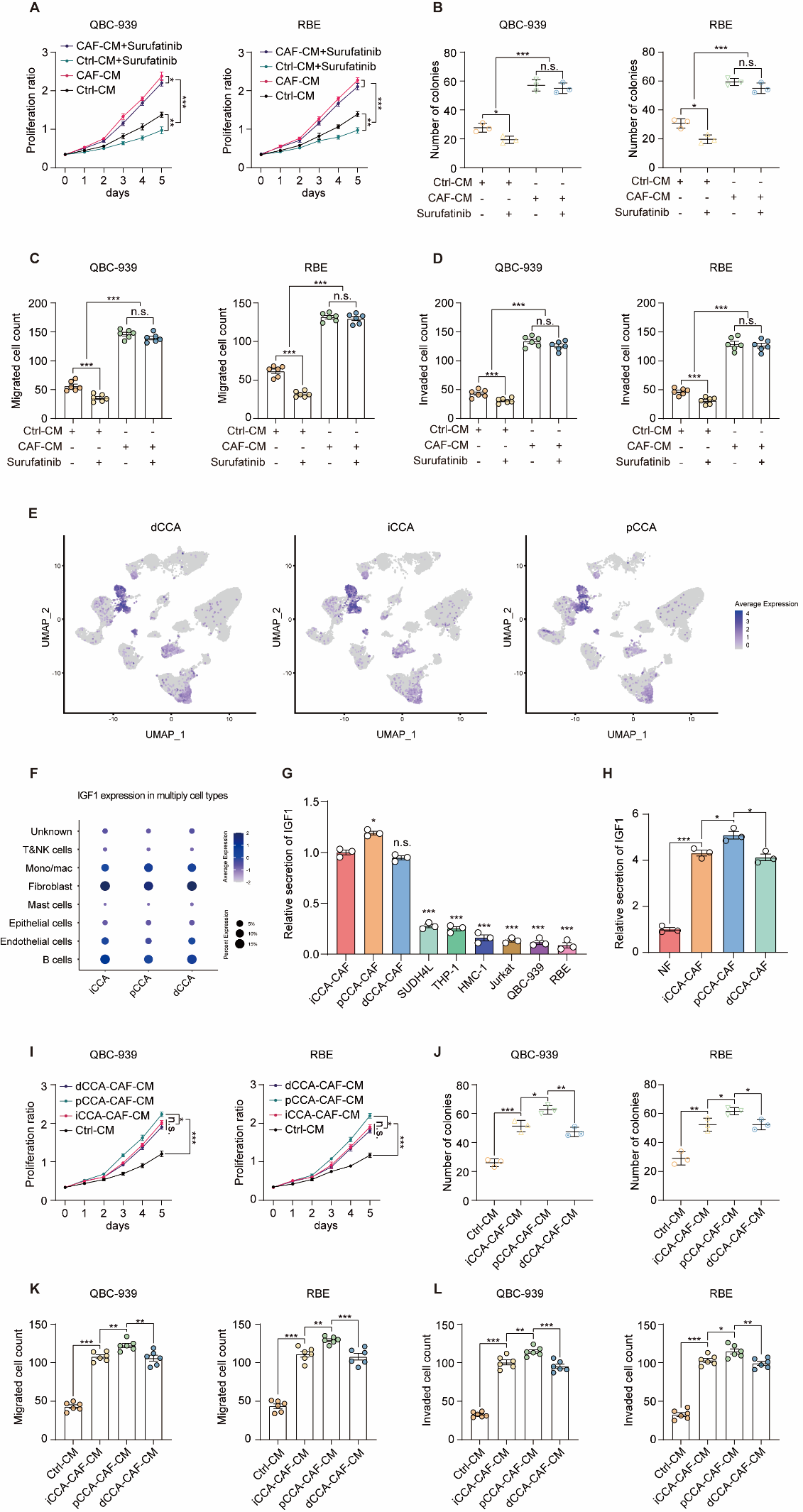


**Supplemental Figure 8. CAF-derived IGF1 promotes malignant phenotypes of cholangiocarcinoma cells.**

(A-D) Functional assays proliferation (A), colony formation (B), migration (C), and invasion (D) evaluating the effects of CAF-CM and surufatinib on CCA cell behavior. (E) UMAP feature plots of IGF1 expression in dCCA, iCCA, and pCCA datasets. (F) Single-cell RNA-seq dot plot showing IGF1 expression level and the percentage of expressing cells across major tumor microenvironment cell types in iCCA/pCCA/dCCA. (G) Relative IGF1 expression across primary CAFs from iCCA, pCCA, and dCCA and the indicated cell lines. (H) Quantification of IGF-1 secretion from NFs and CAFs. (I-L) Effects of CAF-CM from iCCA, pCCA, or dCCA on QBC-939 and RBE cells compared with Ctrl-CM: Proliferation (A), colony formation (B), migration (C), and invasion (D).

Data are from at least three independent experiments and are presented as mean ± SD or representative images. Statistical analyses were performed using unpaired t‑test (B-D, G, H, J-L), and two‑way ANOVA (A, I). Significance indicators: *P < 0.05, **P < 0.01, ***P < 0.001.

**Supplemental Table 1. Correlations between PTPN9 Expression and Clinicopathological Characteristics in CCA**

| **Characteristics** | **Category** | **iCCA** | | | **pCCA** | | | **dCCA** | | |
| --- | --- | --- | --- | --- | --- | --- | --- | --- | --- | --- |
|  |  | **Low** | **High** | **p** | **Low** | **High** | **p** | **Low** | **High** | **p** |
|  |  | n=77 | n=76 |  | n=59 | n=58 |  | n=61 | n=60 |  |
| **Age (years)** | <65 | 45 | 36 | 0.170 | 30 | 36 | 0.221 | 36 | 26 | 0.124 |
|  | ≥65 | 32 | 40 |  | 29 | 22 |  | 26 | 33 |  |
| **Gender** | Male | 44 | 48 | 0.447 | 41 | 39 | 0.794 | 39 | 45 | 0.111 |
|  | Female | 33 | 28 |  | 18 | 19 |  | 23 | 14 |  |
| **Tumor size** | <3cm | 25 | 41 | **0.007** | 27 | 36 | 0.077 | 41 | 43 | 0.420 |
|  | ≥3cm | 52 | 35 |  | 32 | 22 |  | 21 | 16 |  |
| **Differentiation** | High/Moderate | 35 | 43 | 0.168 | 27 | 39 | **0.019** | 26 | 37 | **0.022** |
|  | Low | 42 | 33 |  | 32 | 19 |  | 36 | 22 |  |
| **T stage** | T1/T2 | 50 | 52 | 0.647 | 37 | 47 | **0.027** | 28 | 22 | 0.379 |
|  | T3/T4 | 27 | 24 |  | 22 | 11 |  | 34 | 37 |  |
| **N stage** | N0 | 25 | 18 | 0.227 | 27 | 34 | 0.164 | 42 | 46 | 0.207 |
|  | N1/N2 | 57 | 53 |  | 32 | 24 |  | 20 | 13 |  |
| **Nerve invasion** | Negative | 47 | 54 | 0.191 | 30 | 35 | 0.301 | 29 | 30 | 0.654 |
|  | Positive | 30 | 22 |  | 29 | 23 |  | 33 | 29 |  |

***Continued:***

| **Characteristics** | **Category** | **iCCA** | | | **pCCA** | | | **dCCA** | | |
| --- | --- | --- | --- | --- | --- | --- | --- | --- | --- | --- |
|  |  | **Low** | **High** | **p** | **Low** | **High** | **p** | **Low** | **High** | **p** |
|  |  | n=77 | n=76 |  | n=59 | n=58 |  | n=61 | n=60 |  |
| **MVI** | Negative | 52 | 53 | 0.769 | 46 | 45 | 0.961 | 45 | 46 | 0.493 |
|  | Positive | 25 | 23 |  | 13 | 13 |  | 17 | 13 |  |

**Supplemental Table 2. Univariate and Multivariate Cox Regression Analyses of PTPN9, and Clinicopathological Variables in CCA Patients**

| **Characteristics** | iCCA | | | | pCCA | | | | | dCCA | | | | |  |
| --- | --- | --- | --- | --- | --- | --- | --- | --- | --- | --- | --- | --- | --- | --- | --- |
|  | Univariate analysis | | Multivariate analysis | | | Univariate analysis | | Multivariate analysis | | | Univariate analysis | | Multivariate analysis | | |
|  | **HR** | **p** | **HR** | **p** | | **HR** | **p** | **HR** | **p** | | **HR** | **p** | **HR** | **p** | |
| Age (years) | 0.999 | 0.966 |  |  | | 1.016 | 0.272 |  |  | | 0.989 | 0.462 |  |  | |
| Gender |  |  |  |  | |  |  |  |  | |  |  |  |  | |
| Female | Ref. |  |  |  | | Ref. |  |  |  | | Ref. |  |  |  | |
| Male | 0.805 | 0.327 |  |  | | 0.843 | 0.573 |  |  | | 0.691 | 0.207 |  |  | |
| Tumor size | 1.040 | 0.161 |  |  | | 1.402 | **0.009** |  |  | | 1.192 | 0.210 |  |  | |
| Differentiation |  |  |  |  | |  |  |  |  | |  |  |  |  | |
| High/Moderate | Ref. |  |  |  | | Ref. |  |  |  | | Ref. |  |  |  | |
| Low | 1.947 | **0.003** | 1.754 | **0.025** | | 2.340 | **0.006** |  |  | | 2.468 | **0.002** | 2.119 | 0.033 | |
| T stage |  |  |  |  | |  |  |  |  | |  |  |  |  | |
| T1/T2 | Ref. |  |  |  | | Ref. |  |  |  | | Ref. |  |  |  | |
| T3/T4 | 1.810 | **0.008** | 1.785 | **0.032** | | 2.149 | **0.014** |  |  | | 2.656 | **0.012** |  |  | |
| N stage |  |  |  |  | |  |  |  |  | |  |  |  |  | |
| N0 | Ref. |  |  |  | | Ref. |  |  |  | | Ref. |  |  |  | |
| N1/N2 | 0.873 | 0.573 |  |  | | 2.232 | **0.012** |  |  | | 2.183 | **0.005** |  |  | |

***Continued:***

| **Characteristics** | | iCCA | | | | pCCA | | | | | dCCA | | | | |
| --- | --- | --- | --- | --- | --- | --- | --- | --- | --- | --- | --- | --- | --- | --- | --- |
|  | Univariate analysis | | | Multivariate analysis | | | Univariate analysis | | Multivariate analysis | | | Univariate analysis | | Multivariate analysis | |
|  | **HR** | | **p** | **HR** | **p** | | **HR** | **p** | **HR** | **p** | | **HR** | **p** | **HR** | **p** |
| **Nerve invasion** |  | |  |  |  | |  |  |  |  | |  |  |  |  |
| Negative | Ref. | |  |  |  | | Ref. |  |  |  | | Ref. |  |  |  |
| Positive | 1.450 | | 0.105 |  |  | | 1.651 | 0.084 |  |  | | 1.456 | 0.196 |  |  |
| **MVI** |  | |  |  |  | |  |  |  |  | |  |  |  |  |
| Negative | Ref. | |  |  |  | | Ref. |  |  |  | | Ref. |  |  |  |
| Positive | 1.677 | | **0.020** |  |  | | 2.004 | **0.024** |  |  | | 2.841 | **<0.001** | 2.782 | **0.004** |
| **PTPN9** |  | |  |  |  | |  |  |  |  | |  |  |  |  |
| Low | Ref. | |  |  |  | | Ref. |  |  |  | | Ref. |  |  |  |
| High | 0.371 | | **<0.001** | 0.396 | **<0.001** | | 0.331 | **<0.001** | 0.371 | **0.002** | | 0.386 | **0.001** | 0.374 | **0.005** |

**Supplemental Table 3. Correlations between IGF1R Expression and Clinicopathological Characteristics in CCA**

| **Characteristics** | **Category** | **iCCA** | | | | | | **pCCA** | | | | **dCCA** | | |
| --- | --- | --- | --- | --- | --- | --- | --- | --- | --- | --- | --- | --- | --- | --- |
|  |  | **Low** | | **High** | **p** | **Low** | | | **High** | **p** | **Low** | | **High** | **p** |
|  |  | n=77 | | n=76 |  | | n=59 | | n=58 |  | n=61 | | n=60 |  |
| Age (years) | <65 | | 39 | 42 | 0.057 | | | 31 | 35 | 0.395 | | 30 | 32 | 0.648 |
|  | ≥65 | 38 | | 34 |  | | | 28 | 23 |  | | 31 | 28 |  |
| Gender | Male | 46 | | 46 | 0.921 | | | 44 | 36 | 0.146 | | 41 | 43 | 0.595 |
|  | Female | 31 | | 30 |  | | | 15 | 22 |  | | 20 | 17 |  |
| Tumor size | <3 cm | 36 | | 30 | 0.363 | | | 31 | 32 | 0.775 | | 45 | 39 | 0.295 |
|  | ≥3 cm | 41 | | 46 |  | | | 28 | 26 |  | | 16 | 21 |  |
| Differentiation | High/Moderate | 43 | | 35 | 0.226 | | | 40 | 26 | **0.012** | | 38 | 25 | **0.023** |
|  | Low | 34 | | 41 |  | | | 19 | 32 |  | | 23 | 35 |  |
| T stage | T1/T2 | 50 | | 52 | 0.647 | | | 45 | 39 | 0.278 | | 26 | 24 | 0.770 |
|  | T3/T4 | 27 | | 24 |  | | | 14 | 19 |  | | 35 | 36 |  |
| N stage | N0 | 20 | | 23 | 0.556 | | | 40 | 21 | **0.001** | | 44 | 44 | 0.882 |
|  | N1/N2 | 57 | | 53 |  | | | 19 | 37 |  | | 17 | 16 |  |
| Nerve invasion | Negative | 57 | | 44 | **0.035** | | | 34 | 31 | 0.649 | | 34 | 25 | 0.122 |
|  | Positive | 20 | | 32 |  | | | 25 | 27 |  | | 27 | 35 |  |
| MVI | Negative | 59 | | 46 | **0.032** | | | 48 | 43 | 0.348 | | 50 | 41 | 0.082 |
|  | Positive | 18 | | 30 |  | | | 11 | 15 |  | | 11 | 19 |  |

**Supplemental Table 4. Univariate and Multivariate Cox Regression Analyses of IGF1R, PTPN9, and Clinicopathological Variables in CCA Patients**

| **Characteristics** | iCCA | | | | pCCA | | | | | dCCA | | | | |  |
| --- | --- | --- | --- | --- | --- | --- | --- | --- | --- | --- | --- | --- | --- | --- | --- |
|  | Univariate analysis | | Multivariate analysis | | | Univariate analysis | | Multivariate analysis | | | Univariate analysis | | Multivariate analysis | | |
|  | **HR** | **p** | **HR** | **p** | | **HR** | **p** | **HR** | **p** | | **HR** | **p** | **HR** | **p** | |
| Age (years) | 0.999 | 0.966 |  |  | | 1.016 | 0.272 |  |  | | 0.989 | 0.462 |  |  | |
| Gender |  |  |  |  | |  |  |  |  | |  |  |  |  | |
| Female | Ref. |  |  |  | | Ref. |  |  |  | | Ref. |  |  |  | |
| Male | 0.805 | 0.327 |  |  | | 0.843 | 0.573 |  |  | | 0.691 | 0.207 |  |  | |
| Tumor size | 1.040 | 0.161 |  |  | | 1.402 | **0.009** |  |  | | 1.192 | 0.210 |  |  | |
| Differentiation |  |  |  |  | |  |  |  |  | |  |  |  |  | |
| High/Moderate | Ref. |  |  |  | | Ref. |  |  |  | | Ref. |  |  |  | |
| Low | 1.947 | **0.003** | 1.669 | **0.044** | | 2.340 | **0.006** |  |  | | 2.468 | **0.002** |  |  | |
| T stage |  |  |  |  | |  |  |  |  | |  |  |  |  | |
| T1/T2 | Ref. |  |  |  | | Ref. |  |  |  | | Ref. |  |  |  | |
| T3/T4 | 1.810 | **0.008** | 1.921 | **0.015** | | 2.149 | **0.014** |  |  | | 2.656 | **0.012** |  |  | |
| N stage |  |  |  |  | |  |  |  |  | |  |  |  |  | |
| N0 | Ref. |  |  |  | | Ref. |  |  |  | | Ref. |  |  |  | |
| N1/N2 | 0.873 | 0.573 |  |  | | 2.232 | **0.012** |  |  | | 2.183 | **0.005** |  |  | |
| Nerve invasion |  |  |  |  | |  |  |  |  | |  |  |  |  | |
| Negative | Ref. |  |  |  | | Ref. |  |  |  | | Ref. |  |  |  | |
| Positive | 1.450 | 0.105 |  |  | | 1.651 | 0.084 |  |  | | 1.456 | 0.196 |  |  | |
| MVI |  |  |  |  | |  |  |  |  | |  |  |  |  | |
| Negative | Ref. |  |  |  | | Ref. |  |  |  | | Ref. |  |  |  | |
| Positive | 1.677 | **0.020** |  |  | | 2.004 | **0.024** |  |  | | 2.841 | **<0.001** | 2.482 | **0.009** | |
| IGF1R |  |  |  |  | |  |  |  |  | |  |  |  |  | |
| Low | Ref. |  |  |  | | Ref. |  |  |  | | Ref. |  |  |  | |
| High | 3.039 | **<0.001** | 2.396 | **0.002** | | 3.241 | **<0.001** | 2.818 | **0.002** | | 3.121 | **<0.001** | 2.815 | **0.007** | |
| PTPN9 |  |  |  |  | |  |  |  |  | |  |  |  |  | |
| Low | Ref. |  |  |  | | Ref. |  |  |  | | Ref. |  |  |  | |
| High | 0.371 | **<0.001** | 0.576 | **0.044** | | 0.331 | **<0.001** | 0.465 | **0.022** | | 0.386 | **0.001** | 0.453 | **0.026** | |

**Supplemental Table 5.** **Crystallographic data and refinement statistics.**

| **Data collection** | **PTPN9**  **IGF1R pY^1165/1166^ peptide** |
| --- | --- |
| Space group | P1 |
| Cell dimensions |  |
| a (°A) | 52.669 |
| b (°A) | 74.288 |
| c (°A) | 84.703 |
| a (deg) | 90.052 |
| b (deg) | 86.032 |
| c (deg) | 94.163 |
| Resolution (°A) | 50–2.07 (2.07–2.00) ^a^ |
| Unique observations | 84,400 (8326) ^a^ |
| Completeness (%) | 97.7 (96.3) ^a^ |
| Redundancy | 2.0 (1.9) ^a^ |
| <I>/<σ> | 15.8 (2.057) ^a^ |
| *R*merge | 0.038 (0.247) ^a^ |
| Structure refinement | 36.77–2.00 |
| Resolution (°A) | 2.00 |
| Reflections used for  *R*work/*R*free | 79491 |
| *R*_work_ /*R b* (%) free | 0.2191/0.2578 |
| RMSD ideal bonds (°A) | 0.008 |
| RMSD ideal angles (deg) | 1.17 |
| Ramachandran plot (%) |  |
| Most favored | 95.9 |
| Generously allowed | 1.39 |
| Disallowed | 1.2 |

The values in parentheses correspond to the highest resolution shell.

^a^Each dataset was collected from a single crystal.

**Supplemental Table 6. CCA cohort**

| **Characteristics** | **iCCA (n = 153)** | **pCCA (n = 117)** | **dCCA (n = 121)** |
| --- | --- | --- | --- |
| **Gender** |  |  |  |
| Male | 92 (60.13%) | 80 (68.38%) | 84 (69.42%) |
| Female | 61 (39.87%) | 37 (31.62%) | 37 (30.58%) |
| **Age (years)** | 62.85 ± 9.76 | 62.44 ± 9.99 | 63.19 ± 9.52 |
| **OS time (months)** | 15.00 (9.00–28.00) | 10.00 (6.00–15.00) | 12.00 (8.00–19.00) |
| **OS status** |  |  |  |
| 1 | 85 (55.56%) | 49 (41.88%) | 53 (43.80%) |
| 0 | 68 (44.44%) | 68 (58.12%) | 68 (56.20%) |
| **T stage** |  |  |  |
| 1 | 62 (40.52%) | 30 (25.64%) | 31 (25.62%) |
| 2 | 40 (26.14%) | 54 (46.15%) | 19 (15.70%) |
| 3 | 51 (33.33%) | 33 (28.21%) | 71 (58.68%) |
| **N stage** |  |  |  |
| 0 | 43 (28.10%) | 61 (52.14%) | 88 (72.73%) |
| 1 | 110 (71.90%) | 43 (36.75%) | 26 (21.49%) |
| 2 | 0 (0.00%) | 13 (11.11%) | 7 (5.79%) |
| **AJCC Stage** |  |  |  |
| 1 | 8 (5.23%) | 23 (19.66%) | 29 (23.97%) |
| 2 | 15 (9.80%) | 31 (26.50%) | 85 (70.25%) |
| 3 | 130 (84.97%) | 50 (42.74%) | 7 (5.79%) |
| 4 | 0 (0.00%) | 13 (11.11%) | 0 (0.00%) |
| **Nerve invasion** |  |  |  |
| Positive | 52 (33.99%) | 52 (44.44%) | 62 (51.24%) |
| Negative | 101 (66.01%) | 65 (55.56%) | 59 (48.76%) |
| **MVI** |  |  |  |
| Positive | 48 (31.37%) | 26 (22.22%) | 30 (24.79%) |
| Negative | 105 (68.63%) | 91 (77.78%) | 91 (75.21%) |
| **Differentiation** |  |  |  |
| Moderate | 66 (43.14%) | 56 (47.86%) | 53 (43.80%) |
| Low | 75 (49.02%) | 51 (43.59%) | 58 (47.93%) |
| High | 12 (7.84%) | 10 (8.55%) | 10 (8.26%) |
| **Tumor size (cm)** | 4.50 (2.00–7.40) | 2.50 (2.00–3.50) | 2.10 (1.80–3.00) |

Data are presented as n (%). **MVI**, Microvascular invasion; **AJCC**, American Joint Committee on Cancer. **Tumor size** and **OS** time are summarized as median (IQR).

**Supplemental Table 7. CCA vs. normal tissue cohort**

| **Characteristics** | **iCCA (n = 19)** | **pCCA (n = 18)** | **dCCA (n = 30)** |
| --- | --- | --- | --- |
| **Age** | 58.74 ± 7.23 | 62.17 ± 6.96 | 61.63 ± 10.30 |
| **Gender** |  |  |  |
| Male | 13 (68.42%) | 11 (61.11%) | 23 (76.67%) |
| Female | 6 (31.58%) | 7 (38.89%) | 7 (23.33%) |
| **Tumor size** | 3.70 (2.55–5.15) | 2.95 (2.10–3.58) | 2.35 (1.73–3.05) |
| **T stage** |  |  |  |
| 1 | 12 (63.16%) | 4 (22.22%) | 6 (20.00%) |
| 2 | 4 (21.05%) | 14 (77.78%) | 15 (50.00%) |
| 3 | 3 (15.79%) | 0 (0.00%) | 9 (30.00%) |
| 4 | 0 (0.00%) | 0 (0.00%) | 0 (0.00%) |
| **N stage** |  |  |  |
| 0 | 15 (78.95%) | 11 (61.11%) | 24 (80.00%) |
| 1 | 4 (21.05%) | 6 (33.33%) | 6 (20.00%) |
| 2 | 0 (0.00%) | 1 (5.56%) | 0 (0.00%) |
| **AJCC stage** |  |  |  |
| 1 | 8 (42.11%) | 2 (11.11%) | 5 (16.67%) |
| 2 | 4 (21.05%) | 9 (50.00%) | 25 (83.33%) |
| 3 | 7 (36.84%) | 6 (33.33%) | 0 (0.00%) |
| 4 | 0 (0.00%) | 1 (5.56%) | 0 (0.00%) |
| **Nerve invasion** |  |  |  |
| Positive | 5 (26.32%) | 15 (83.33%) | 21 (70.00%) |
| Negative | 14 (73.68%) | 3 (16.67%) | 9 (30.00%) |
| **MVI** |  |  |  |
| Positive | 5 (26.32%) | 10 (55.56%) | 12 (40.00%) |
| Negative | 14 (73.68%) | 8 (44.44%) | 18 (60.00%) |
| **Differentiation** |  |  |  |
| Moderate | 6 (31.58%) | 8 (44.44%) | 11 (36.67%) |
| Low | 12 (63.16%) | 9 (50.00%) | 16 (53.33%) |
| High | 1 (5.26%) | 1 (5.56%) | 3 (10.00%) |

Data are presented as n (%). **MVI**, Microvascular invasion; **AJCC**, American Joint Committee on Cancer. **Tumor size** is summarized as median (IQR).

**Supplemental Table 8. Surufatinib treatment cohort**

| **Characteristics** | **All patients (n = 24)** |
| --- | --- |
| **Gender** |  |
| Male | 14 (58.33%) |
| Female | 10 (41.67%) |
| **Age (years)** | 61.29 ± 10.24 |
| **T** |  |
| 2 | 19 (79.17%) |
| 3 | 3 (12.50%) |
| 4 | 2 (8.33%) |
| **N** |  |
| 0 | 7 (29.17%) |
| 1 | 17 (70.83%) |
| **M** |  |
| 0 | 19 (79.17%) |
| 1 | 5 (20.83%) |
| **AJCC Stage** |  |
| Ⅱ | 6 (25.00%) |
| Ⅲ | 13 (54.17%) |
| Ⅳ | 5 (20.83%) |
| **Tumor size** | 6.55 (5.40–8.68) |

Data are presented as n (%). **AJCC**, American Joint Committee on Cancer. **Tumor size** is summarized as median (IQR).

**Supplemental Table 9. CAF isolation cohort**

| **Characteristics** | **iCCA (n = 12)** | **pCCA (n = 15)** | **iCCA (n = 3)** |
| --- | --- | --- | --- |
| **Gender** |  |  |  |
| Male | 7 (58.33%) | 12 (80.00%) | 2 (66.67%) |
| Female | 5 (41.67%) | 3 (20.00%) | 1 (33.33%) |
| **Age(years)** | 61.00 ± 9.42 | 60.80 ± 9.78 | 65.00 ± 6.68 |
| **T** |  |  |  |
| 1 | 11 (91.67%) | 4 (26.67%) | 3 (100.00%) |
| 2 | 1 (8.33%) | 11 (73.33%) | 0 (00.00%) |
| **N** |  |  |  |
| 0 | 10 (83.33%) | 9 (60.00%) | 2 (66.67%) |
| 1 | 2 (16.67%) | 6 (40.00%) | 1 (33.33%) |
| **AJCC Stage** |  |  |  |
| 1 | 9 (75.00%) | 2 (13.33%) | 3 (100.00%) |
| 2 | 1 (8.33%) | 7 (46.67%) | 0 (00.00%) |
| 3 | 2 (16.67%) | 6 (40.00%) | 0 (00.00%) |
| **Nerve invasion** |  |  |  |
| Positive | 4 (33.33%) | 10 (66.67%) | 3 (100.00%) |
| Negative | 8 (66.67%) | 5 (33.33%) | 0 (0.00%) |
| **MVI** |  |  |  |
| Positive | 3 (25.00%) | 1 (6.67%) | 0 (0.00%) |
| Negative | 9 (75.00%) | 14 (93.33%) | 3 (100.00%) |
| **Differentiation** |  |  |  |
| Low | 7 (58.33%) | 5 (33.33%) | 1 (33.33%) |
| Moderate | 5 (41.67%) | 7 (46.67%) | 2 (66.67%) |
| High | 0 (00.00%) | 3 (20.00%) | 0 (00.00%) |
| **Tumor size** | 5.45 (4.20–6.58) | 2.50 (2.20–3.00) | 1.80 (1.45–2.15) |

Data are presented as n (%). **MVI**, Microvascular invasion; **AJCC**, American Joint Committee on Cancer. **Tumor size** is summarized as median (IQR).
